# Molecular insights into replication initiation in a multipartite genome harboring bacterium *Deinococcus radiodurans*

**DOI:** 10.1016/j.jbc.2021.100451

**Published:** 2021-02-21

**Authors:** Ganesh K. Maurya, Reema Chaudhary, Neha Pandey, Hari S. Misra

**Affiliations:** 1 Molecular Biology Division, Bhabha Atomic Research Centre, Mumbai, India; 2 Life Sciences, Homi Bhabha National Institute, Mumbai, India; 3 Life Sciences, University of Mumbai, Mumbai, India

**Keywords:** ATPase, bacterial genetics, chromosomes, DNA–protein interaction, metabolic regulation, origin of replication, protein–protein interaction, radiation biology, BACTH, bacterial two-hybrid, ChrI, chromosome I, ChrII, chromosome II, CTD, C-terminal deletion, dsDNA, double-stranded DNA, FRET, fluorescence resonance energy transfer, PIR, post irradiation recovery, ssDNA, single-stranded DNA

## Abstract

*Deinococcus radiodurans* harbors a multipartite ploid genome system consisting of two chromosomes and two plasmids present in multiple copies. How these discrete genome elements are maintained and inherited is not well understood. PprA, a pleiotropic protein involved in radioresistance, has been characterized for its roles in DNA repair, genome segregation, and cell division in this bacterium. Here, we show that PprA regulates ploidy of chromosome I and II and inhibits the activity of drDnaA, the initiator protein in *D. radiodurans*. We found that *pprA* deletion resulted in an increased genomic content and ploidy of both the chromosomal elements. Expression of PprA *in trans* rescued the phenotypes of the *pprA* mutant. To understand the molecular mechanism underlying these phenotypes, we characterized drDnaA and drDnaB. As expected for an initiator protein, recombinant drDnaA showed sequence-specific interactions with the putative *oriC* sequence in chromosome I (*oriCI*). Both drDnaA and drDnaB showed ATPase activity, also typical of initiator proteins, but only drDnaB exhibited 5′→3′ dsDNA helicase activity *in vitro*. drDnaA and drDnaB showed homotypic and heterotypic interactions with each other, which were perturbed by PprA. Interestingly, PprA has inhibited the ATPase activity of drDnaA but showed no effect on the activity of drDnaB. Regulation of chromosome copy number and inhibition of the initiator protein functions by PprA strongly suggest that it plays a role as a checkpoint regulator of the DNA replication initiation in *D. radiodurans* perhaps through its interaction with the replication initiation machinery.

The origin of replication in bacterial chromosome (*oriC*) is a discrete locus that contains AT-rich conserved DNA motifs and a varying number of 9 mer repeats of nonpalindromic sequences called DnaA boxes. These boxes are recognized by a replication initiator protein named DnaA, followed by the assembly of replication initiation complex at *oriC* (1, 2). Mechanisms underlying replication initiation have been characterized in large number of bacteria harboring limited copies of single circular chromosome as inheritable genetic material (3, 4). In *Escherichia coli*, it has been shown that DnaA-ATP oligomer binds to DnaA boxes at *oriC* and unwinds the adjacent AT-rich region. Subsequently, a hexameric complex of replicative helicase DnaB and its loader DnaC (DnaB_6_-DnaC_6_) is recruited to the unwound region in *oriC* resulting in the formation of the prepriming complex (5, 6). This provides the site for binding of primase and activation of various events required for the progression of the replication complex that includes DNA polymerase III holoenzyme. DnaB hexameric ring translocates bidirectionally to unwind the parental duplex DNA while the 3′ end of the primer is extended by DNA polymerase complex (7, 8). In *E. coli*, the initiation of DNA replication at *oriC* site is tightly regulated and the next round of *oriC*-mediated DNA replication must have to wait till the *oriC* of newly replicated daughter chromosomes is fully methylated. Therefore, under normal growth conditions, the number of copies of the primary chromosome per cell is expected to be less than 2 (5). Recently, the bacteria with multiple copies of the multipartite genome system have been reported. Notably, most of them are either parasites to some forms of life or exhibit super tolerance to abiotic stresses (9). The ploidy of chromosomes in these bacteria allowed us to revisit the mechanism of *oriC* regulation as known in bacteria containing less than two copies of circular chromosome per cell. Mechanisms underlying the regulation of *oriC* function in multipartite genome harboring bacteria have not been studied in detail. In the case of *Vibrio cholerae*, which harbors two chromosomes, namely chromosome I (Chr I) and chromosome II (Chr II), the Chr I replicates similar to the *E. coli* chromosome while Chr II follows a replication mechanism that is akin to low copy number P1 and F plasmids (10, 11, 12, 13, 14, 15).

*Deinococcus radiodurans* is characterized for its extraordinary resistance to the lethal doses of DNA-damaging agents including radiation and desiccation (16, 17, 18, 19). It harbors a multipartite genome system comprised of two chromosomes (Chr I (2,648,638 bp) and Chr II (412,348 bp)) and a megaplasmid (177,466 bp) and a plasmid (45,704 bp) (20). Interestingly, each genome element is present in multiple copies per cell (21). Chr I of *D. radiodurans* encodes the putative DnaA (DR_0002) and DnaB (DR_0549) (hereafter named drDnaA and drDnaB, respectively) while chromosome II encodes PprA (DR_A0346), which has been characterized for various functions (22, 23, 24, 25). Recently, the extended structure of PprA has been reported where a possibility of it acting as a protein scaffold has been suggested (26). Here, for the first time, we report the functional characterization of chromosome replication initiation proteins drDnaA and drDnaB in *D. radiodurans* and demonstrate that PprA plays an important role in regulation of DNA replication. The *pprA* mutant showed an increased copy number of chromosome I (ChrI) and chromosome II (ChrII), which was complemented by the *in trans* expression of the wild-type PprA. Interestingly, there was no effect of PprA over expression on copy number of the different genome elements in the wild-type cells. drDnaA was characterized as a sequence-specific *origin of replication* (*oriCI*) binding protein and an *oriCI* responsive ATPase. drDnaB was found to be an ATP-dependent 5′→3′ dsDNA helicase and showed higher affinity for single-stranded DNA (ssDNA) than double-stranded DNA (dsDNA). Interestingly, PprA interacted with drDnaA at a relatively higher affinity than drDnaB and inhibited both homotypic and heterotypic interactions of these proteins. Further, PprA downregulated the ATPase activity of drDnaA but showed no effect on ATPase and helicase activities of drDnaB. These results suggest that drDnaA and drDnaB carry out the necessary functions required for initiation of replication at *oriCI* in *D. radiodurans*. Furthermore, the interference imposed by PprA in the physicochemical properties of these replication proteins as well as an increase in the copy numbers of both primary and secondary chromosomes in its absence together suggested the involvement of PprA in regulation of chromosomal replication in this bacterium.

## Results

### The pprA deletion affects genomic content in *D. Radiodurans*

**Figure 1:**
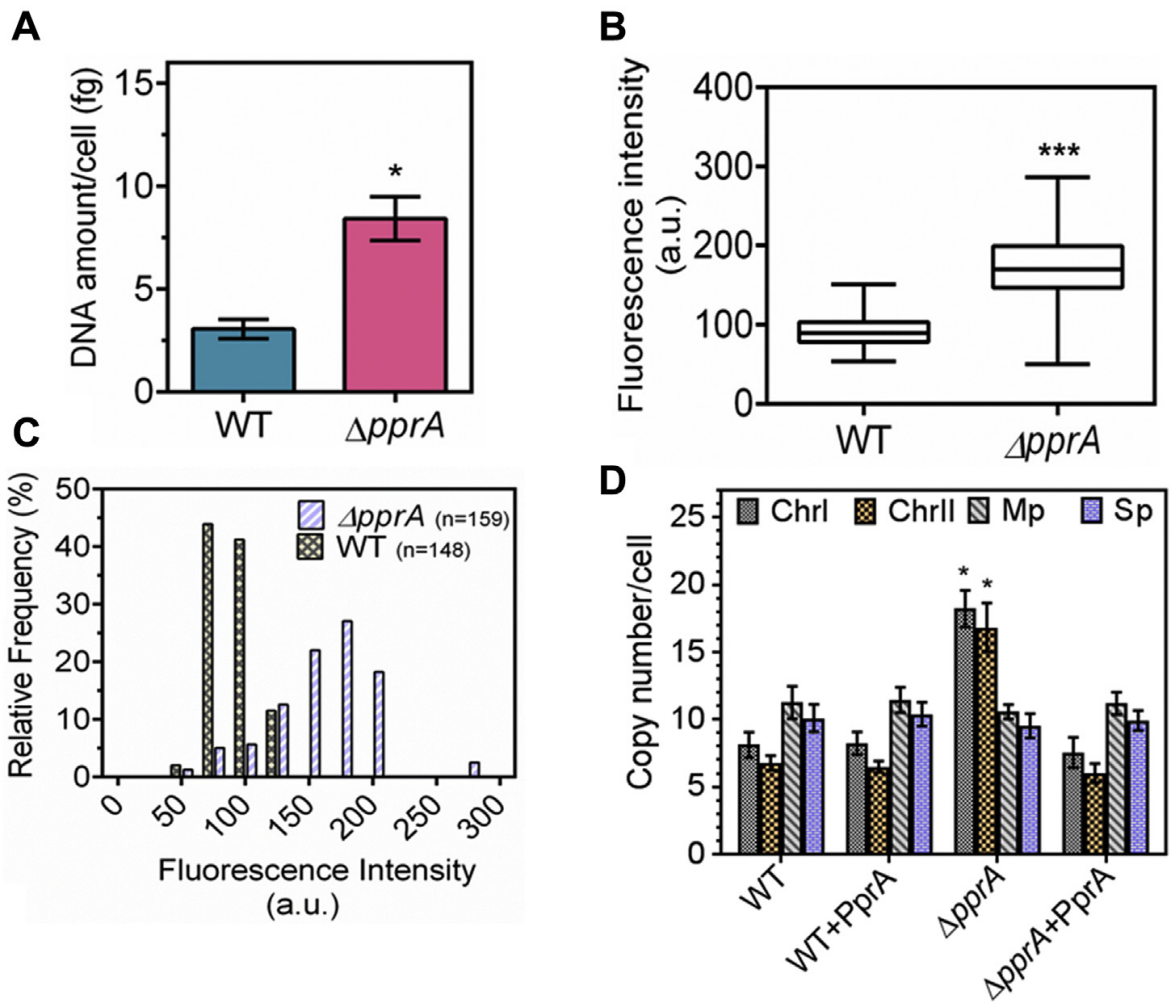
**Change in genomic content in *pprA* deletion mutant of *D. radiodurans*.***D. radiodurans* R1 (WT) and its *pprA* mutant (Δ*pprA*) were grown to mid-logarithmic phase. The amount of DNA per cell (fg; femtograms) was measured spectrophotometrically (*A*) and through DAPI staining of nucleoid (*B*). The changes in DAPI fluorescence in wild type and Δ*pprA* mutant cells were scanned in ∼150 cells and relative frequency of cells containing different amounts of DAPI stained nucleoid was estimated (*C*). Similarly, wild type (WT) and mutant (Δ*pprA*) populations were expressed with PprA *in trans* (WT + PprA, Δ*pprA* + PprA) and the copy number of chromosome I (ChrI), chromosome II (Chr II), megaplasmid (Mp), and small plasmid (Sp) was determined (*D*). Data presented in *A*, *B*, and *D* are mean ± SD (n = 9). Data given in *C* are from the wild-type cell population (n = 148) and mutant population (n = 159). Data was analyzed by Student’s *t*-test and *p* values less than 0.5 and 0.001 were denoted as (∗) and (∗∗∗), respectively.

Earlier, the regulatory role of PprA in cell division and genome maintenance has been demonstrated (23, 24, 27, 28). In this study, the DNA content and the copy number of genome elements in *pprA* deletion mutant were compared with the wild-type *D. radiodurans*. The total DNA content in mutant cells (∼8.41 ± 1.05 fg per cell) was found to be approximately threefold higher than wild-type cells (∼3.05 ± 0.47 fg per cell) (Fig. 1*A*). Similarly, the DAPI fluorescence in Δ*pprA* mutant was approximately twofold higher than that of wild type (Fig. 1*B*). The cell scan analysis of DAPI stained cells showed that ∼80% of Δ*pprA* cells have approximately twofold higher DAPI fluorescence as compared with wild-type cells grown identically (Fig. 1*C*). When we checked the copy number of each replicon per cell, we found that the average copy number of Chr I and Chr II was ∼2.5- to 3-fold higher in Δ*pprA* mutant as compared with the wild type. For instance, the average copy number of Chr I was ∼8 per cell in wild type while it was ∼18 per cell in the Δ*pprA* mutant. The average copy number of Chr II also increased from ∼7 in wild type to ∼17 per cell in the Δ*pprA* mutant (Fig. 1*D*). Further we checked the effect of *in trans* expression of PprA on the copy number of these genome elements in wild type and Δ*pprA* mutant. We observed nearly no effect of PprA over expression in wild type, whereas the Δ*pprA* mutant expressing PprA *in trans* could restore the wild-type copies of the genome element (Fig. 1*D*). Interestingly, the copy number of megaplasmid and small plasmid did not change under any of these conditions. These results indicated a possible role of PprA in the maintenance of the chromosome copy number possibly by regulating the replication of DNA in *D. radiodurans*.

### PprA interacts with drDnaA and drDnaB

**Figure 2:**
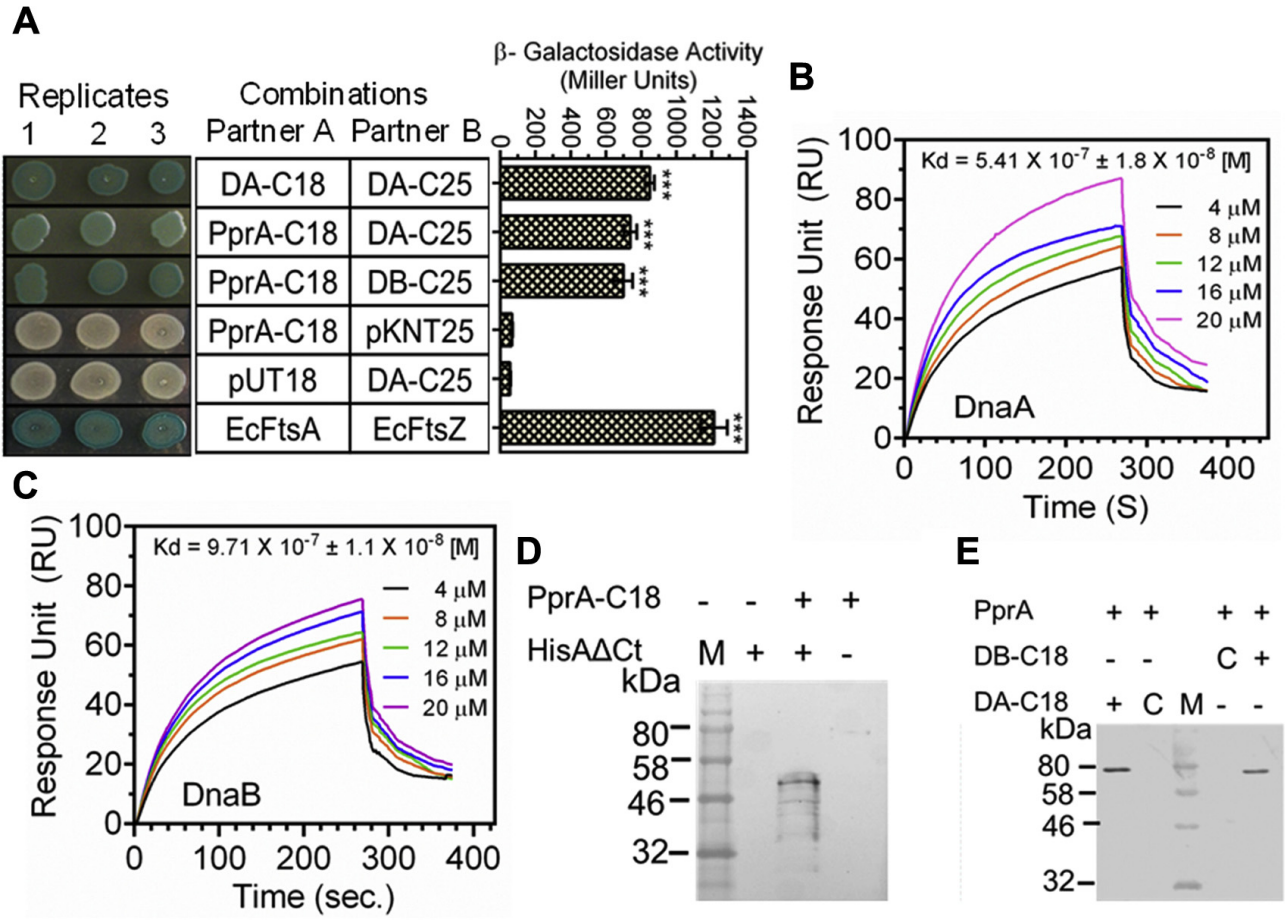
**PprA interaction with drDnaA and drDnaB of *D. radiodurans*.***E. coli* BTH 101 cells coexpressing drDnaA-C18 (DA-C18), drDnaA-C25 (DA-C25), drDnaB-C25 (DB-C25), PprA-C18 in different combinations were detected for expression of β-galactosidase in spot test and liquid (*A*). The bacterial two-hybrid system vectors pUT18 and pKNT25 were used as negative control while *E. coli* FtsA (EcFtsA) and FtsZ (EcFtsZ) were used a positive control. Recombinant PprA interactions with recombinant drDnaA (DnaA) (*B*) and drDnaB (DnaB) (*C*) of *D. radiodurans* were studied by surface plasmon resonance. *Ex vivo* interaction of PprA expressing from PprA-C18 and C-terminal truncated DnaA with histidine tag (HisAΔCt) was monitored in surrogate *E. coli* (*D*). *In vivo* interaction of native PprA expressing on chromosome and drDnaA (DA-C18) and drDnaB (DB-C18) on plasmids was monitored in *D. radiodurans* R1. Total proteins were precipitated with antibodies against polyhistidine (*D*) and PprA (*E*). Perspective interacting partners were detected using antibodies against the T18 domain of CyaA. Sizes of fusions were compared with the molecular weight marker (M). C in panel *E* indicates plasmid control. Data shown in *A* panel was analyzed by Student’s *t*-test and *p* values less than 0.001 were denoted as (∗∗∗), respectively.

To obtain the mechanistic insights into the regulatory role of PprA in DNA replication, the physical and functional interaction of PprA with drDnaA or drDnaB proteins was monitored using bacterial two-hybrid system. For this, T18-tagged PprA and T25-tagged drDnaA or drDnaB were coexpressed in *E. coli* BTH101 (*cyaA*^−^). Interaction of two target proteins tagged with T18 and T25 domains of CyaA would reconstitute the active CyaA resulting in induction of transcription from β-galactosidase gene in BTH101. Expression of β-galactosidase as an indication of protein–protein interaction was monitored using spot test. PprA showed interaction with both drDnaA and drDnaB as indicated by a blue color in the spot test (Fig. 2*A*). The interaction of PprA with these proteins was further checked using surface plasmon resonance (SPR) using purified recombinant drDnaA or drDnaB (Fig. S1). Results showed a concentration-dependent increase in the SPR signals for both drDnaA and drDnaB (Fig. 2, *B* and *C*). The dissociation constant (Kd) for drDnaA was 5.41 × 10^−7^ ± 1.8 X 10^−8^ [M] while it was 9.71 × 10^−7^ ± 1.1 × 10^−8^ [M] for drDnaB indicating that PprA interacts with drDnaA with approximately twofold higher affinity than with drDnaB. Interaction of C-terminal deletion (CTD) mutant of drDnaA (DnaAΔCt) with PprA was tested in surrogate *E. coli* using co-immunoprecipitation. Results showed that the deletion of CTD did not affect drDnaA interaction with PprA (Fig. 2*D*) suggesting that drDnaA might interact with PprA through its N-terminal domain and/or the middle region of the protein. *In vivo* interaction of PprA with drDnaA and dDnaB was tested using co-immunoprecipitation assays with the cellular protein extracts from *D. radiodurans* cells transformed with T18-tagged drDnaA or drDnaB expressing plasmid. Endogenous PprA was precipitated using anti-PprA antibodies and the presence of T18-tagged drDnaA/drDnaB was probed using immunoblotting with T18 antibodies. Consistent with the above results, both drDnaA and drDnaB had co-immunoprecipitated with PprA *in vivo* (Fig. 2*E*). These results together suggested that PprA interacts with both drDnaA and drDnaB of *D. radiodurans*.

### drDnaA is a sequence-specific oriCI binding protein

The putative *origin of replication* of chromosome I (*oriCI*) in *D. radiodurans* R1, spanning 1183 to 1903 bp upstream of *drdnaA* gene (DR_0002), was predicted using the DOriC database (DoriC accession number – ORI10010007) (29). Sequence analysis using WebLogo online tool revealed the presence of consensus sequences of 13 copies of 9-mer DnaA-boxes (30) between 1273 and 1772 bp (∼500 bp) upstream of the dr*dnaA* gene. A 46.2% GC content of *oriCI* indicated an AT-rich sequence, typical of canonical *oriC* sequences. The structure of *oriCI* was found to be different as compared with the *E. coli oriC*. It is comparatively longer than the *E. coli oriC* and contains only eight out of 13 perfect *E. coli* like DnaA boxes (TTATCCACA). Five out of 13 DnaA boxes were imperfect with single nucleotide difference from the *E. coli* type boxes and were similar to other bacteria such as *Cyanothece* 51142, *Thermus thermophilus*, and *Bacillus subtilis* (Fig. S2) (31, 32, 33). A web logo of these DnaA boxes was created yielding a consensus sequence of T(A/G)TA(T)TCCACA. These DnaA boxes are distributed randomly on both sense (four DnaA boxes) and antisense (nine DnaA boxes) strands of chromosome I. This suggested that *oriCI* in chromosome I of *D. radiodurans* is largely similar to *oriC* of *E.coli* with some deviations. Whether these changes have any functional significance, in the context of regulation of replication initiation, needs to be explored.

**Figure 3:**
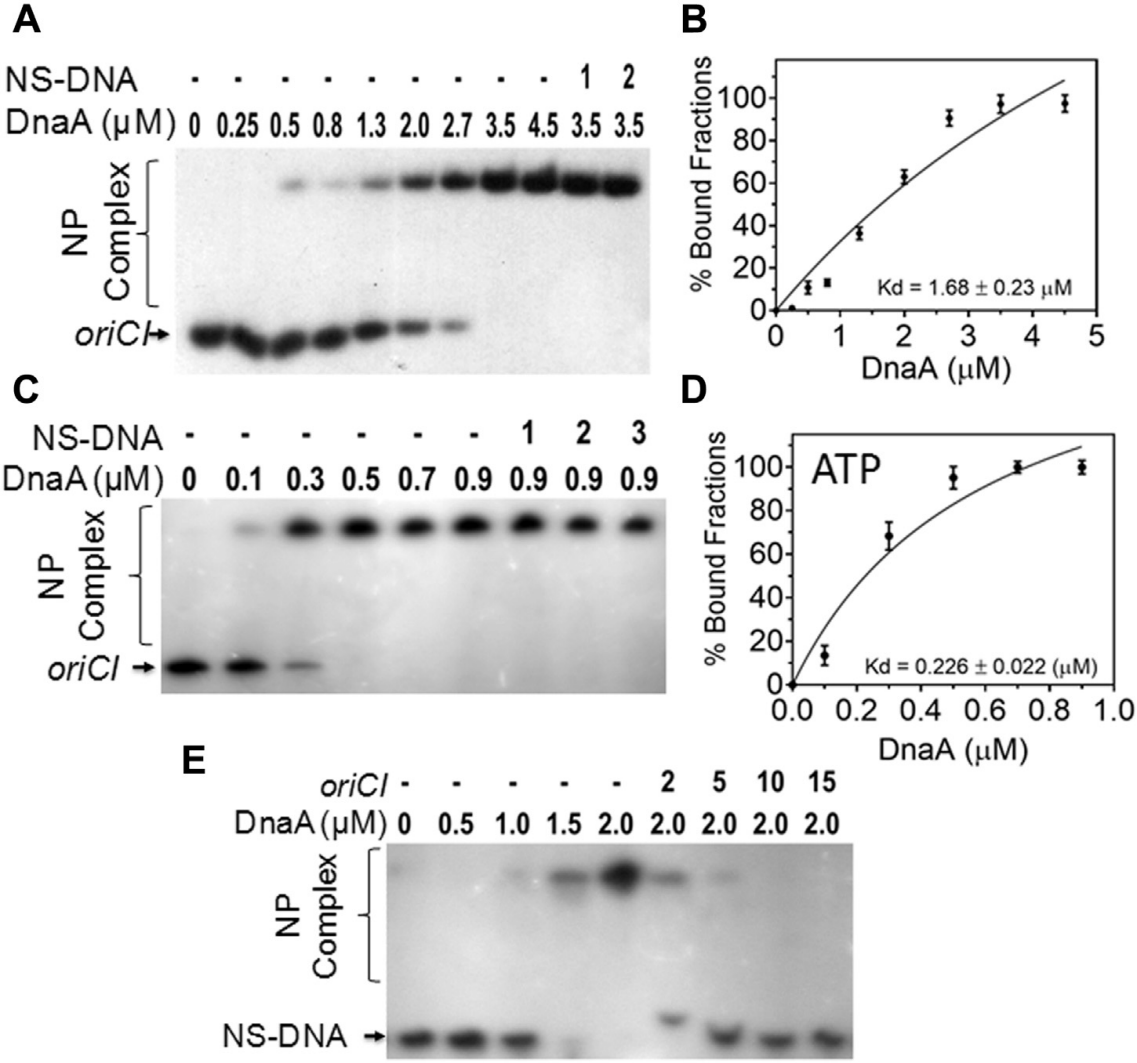
**Recombinant drDnaA interaction with putative *oriCI*.** The linear *oriCI* DNA was radiolabeled and incubated with increasing concentration of recombinant drDnaA protein (DnaA) in the absence (*A* and *B*) and presence (*C* and *D*) of 1 mM ATP and titrated with 5 (1), 10 (2), 20 (3), and 50 (4) fold higher molar concentration of nonspecific dsDNA (NS-DNA). Similarly, an increasing concentration of drDnaA was incubated with linear nonspecific DNA (NS-DNA) and titrated with 2-, 5-, 10-, and 15-fold higher molar concentration of *oriCI* (*E*). The nucleoprotein complexes were analyzed on nondenaturing PAGE. Band intensity of free and bound DNA was estimated densitometrically and plotted as mean ± SD (n = 3) of bound fraction in percentage in *B* and *D*. Data shown in *E* panel is a representative of reproducible experiment repeated three times independently.

Binding of recombinant drDnaA (Fig. S1A) to [^32^P] end labeled *oriCI* containing 13 repeats of DnaA-boxes was checked by electrophoretic mobility shift assays (EMSA). The recombinant drDnaA showed sequence-specific interaction with *oriCI* as the binding did not change even in the presence of 50-fold excess of cold nonspecific DNA (Fig. 3, *A* and *B*). Interestingly, the binding affinity of drDnaA for *oriCI* (Kd = 1.68 ± 0.23 μM) was increased by approximately eightfold in presence of ATP (Kd = 0.243 ± 0.02 μM) (Fig. 3, *C* and *D*). Further, binding of [^32^P] end labeled nonspecific DNA with increasing concentrations of drDnaA was displaced by addition of higher molar concentration of cold *oriCI* DNA confirming the sequence-specific interaction of drDnaA to *oriCI* (Fig. 3*E*). In addition, the binding affinity of drDnaA for *oriCI* decreased upon reducing the number of DnaA boxes (Fig. S3) in the presence of ATP (Table 1). The effect of histidine tag on DNA-binding activity drDnaA was ruled out as both (his)6 tagged and non-(his)6 tagged drDNA showed nearly similar Kd for dsDNA (Fig. S4). Similar to *E. coli* DnaA, which shares 44.4% identity and 72% similarity with drDnaA (data not shown), drDnaA showed specific binding even for the *oriCI* sequence with only a single DnaA box. However, the affinity of drDnaA for a perfect DnaA box (TTATCCACA) was approximately twofold higher (Kd = 2.88 ± 0.28 μM) than the affinity (Kd = 4.21 ± 0.15 μM or 4.36 ± 0.18 μM) for imperfect DnaA boxes (TTTTCCACA or GTATCCACA) (Table 1). These results suggested that drDnaA binds to *oriCI* in a sequence-specific manner and this interaction is stimulated by ATP.

**Table 1:** Dissociation constant (Kd) of drDnaA with *oriCI* and its repeat variants with the different number of DnaA boxes was measured in the presence and absence of ATP

Number of repeats	Length (bp)	ATP	Dissociation constant (Kd) Mean ± SD (μM)
Full length (13 repeats)	500 bp	−	1.78 ± 0.23
Full length (13 repeats)	500 bp	+	0.243 ± 0.021
11 Repeats	261 bp	+	0.241 ± 0.01
7 Repeats	164 bp	+	0.373 ± 0.01
3 Repeats	37 bp	+	1.08 ± 0.12
1 Repeat (TTATCCACA)	16 bp	+	2.88 ± 0.28
1 (TTTTCCACA) nonperfect repeat	16 bp	+	4.21 ± 0.15
1 (GTATCCACA) nonperfect repeat	16 bp	+	4.36 ± 0.18

**Figure 4:**
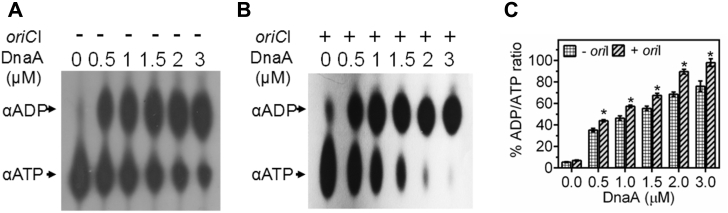
**ATPase activity in recombinant drDnaA.** An increasing concentration (0.5, 1, 1.5, 2.0, and 3 μM) of recombinant drDnaA (DnaA) was incubated with [^32^P]-αATP (αATP) in the absence (*A*) and presence (*B*) of linear *oriCI* (*oriI*) DNA and generation of [^32^P]-αADP (αADP) product was detected on TLC after autoradiography. Spot intensity was quantified densitometrically and percent of ADP/ATP ratios was plotted as a function of drDnaA concentration (*C*). Results were analyzed using Student’s *t*-test and significant difference in data sets with *p* values of 0.05 or less is shown as (∗).

Recombinant purified drDnaA was incubated with radiolabeled repeat variants of linear oriCI in the presence (+) and absence (−) of ATP and EMSA was carried out, and autoradiograms were developed as shown in Figure 4. Fractions of DNA bound to protein were estimated densitometrically and plotted as a function of protein concentration. The Kd for the curve fitting of individual plots was determined using GraphPad Prism6 software.

### C-terminal domain of drDnaA regulates oriCI stimulation of its ATPase activity

**Figure 5:**
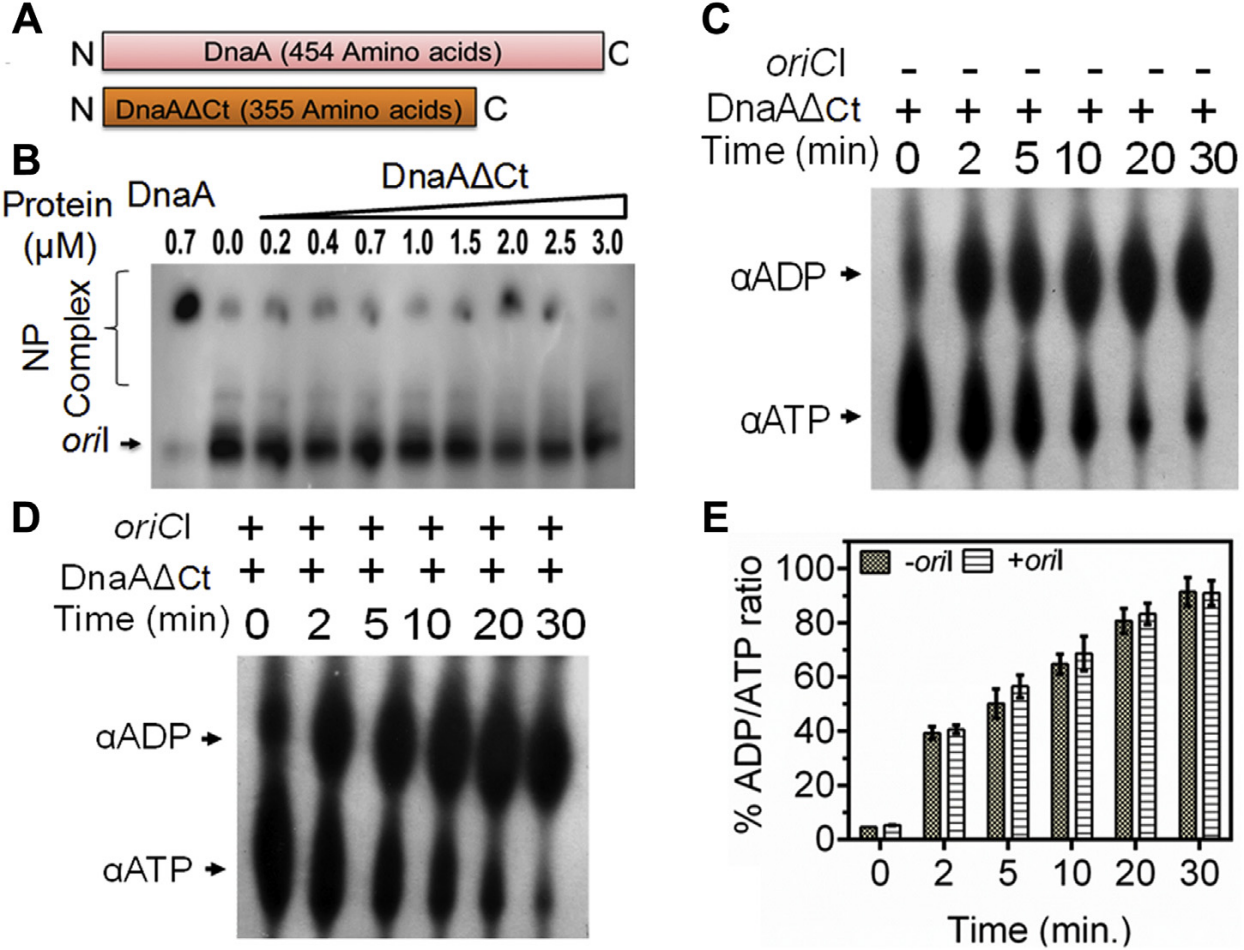
**Role of C-terminal putative helix-turn-helix motifs containing domain in drDnaA function.** The 99 amino acids of drDnaA were removed from its C terminal and resulting DnaAΔCt derivative was generated (*A*). An increasing concentration of recombinant protein was incubated with radiolabeled linear *oriCI* (*oriI*) DNA as described in Figure 4*A*. Products were analyzed on native PAGE and autoradiogram was developed (*B*). Similarly, 2 μM concentration of DnaAΔCt was incubated for different time points with [^32^P]-αATP in the absence (*C*) and presence (*D*) of linear *oriCI* (*oriI*) DNA. Products were separated on TLC and analyzed as described in Figure 4. The percentage of ADP/ATP ratios was plotted as a function of time (*E*). Results were analyzed using Student’s *t*-test and significant difference in data sets with *p* values of 0.05 or less is shown as (∗).

Since, drDnaA showed a relatively higher affinity for *oriCI* in the presence of ATP (Fig. 3*C*), ATP hydrolysis by drDnaA in the presence and absence of *oriCI* was tested. Results showed that drDnaA hydrolyzes [^32^P] αATP into [^32^P] αADP (Fig. 4*A*). Interestingly, the ATP hydrolysis by drDnaA is stimulated in the presence of *oriCI* (Fig. 4, *B* and *C*) but not in the presence of nonspecific dsDNA (Fig. S5). The helix-turn-helix motif in domain IV at the C terminal of DnaA is known to confer sequence specificity for DnaA–*oriC* interaction in many bacteria (34, 35, 36, 37, 38, 39). Therefore, the binding of drDnaA having a deleted C terminal (domain IV) (DnaAΔCt) (Fig. 5*A*) with *oriCI* was checked in the presence and absence of ATP. DnaAΔCt failed to bind *oriCI* (Fig. 5*B*) and the ATPase activity of DnaAΔCt (Fig. 5*C*) did not change in the presence of *oriCI* (Fig. 5, *D* and *E*). Further, the effect of CTD deletion on rate of ATP hydrolysis was calculated using both drDnaA and DnaAΔCt proteins at 30 nM [^32^P] αATP. The rate of ATP hydrolysis in drDnaA (0.76 ± 0.04 nM/min) was found to be nearly similar to DnaAΔCt (0.81 ± 0.05 nM/min). These results suggested that the C-terminal domain IV of drDnaA is essential for its binding to *oriCI* and thus for *oriCI*-dependent stimulation of ATPase function but seems to have no role in the ATPase activity of drDnaA *per se*.

### drDnaB is an ssDNA-responsive ATPase and an ATP-dependent 5′→3′ dsDNA helicase

**Figure 6:**
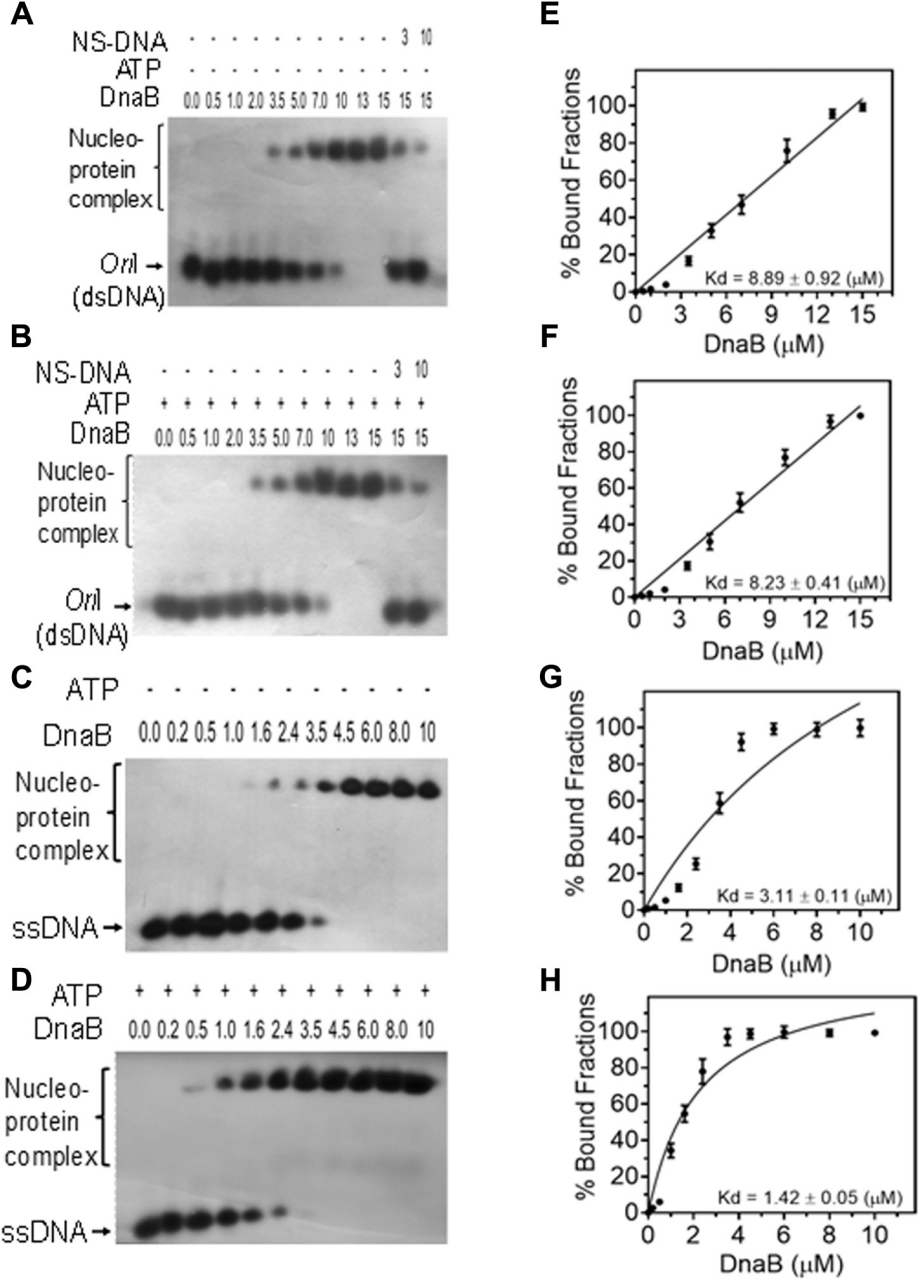
**DNA binding activity of DnaB of *D. radiodurans* (drDnaB).** An increasing concentration (μM) of recombinant purified drDnaB (DnaB) was incubated with radiolebeled linear *oriCI* (*oriI*) taken as dsDNA (*A* and *B*) and radiolabeled ssDNA (*C* and *D*) in the absence (*A* and *C*) and presence (*B* and *D*) of ATP. Products were analyzed on nondenaturing PAGE. Autoradiograms were developed, band intensity of free and bound form of DNA was estimated densitometrically from autoradiogram *A* (*E*), *B* (*F*), *C* (*G*), and *D* (*H*), and the mean ± SD (n = 3) of percent bound fraction was plotted.

**Figure 7:**
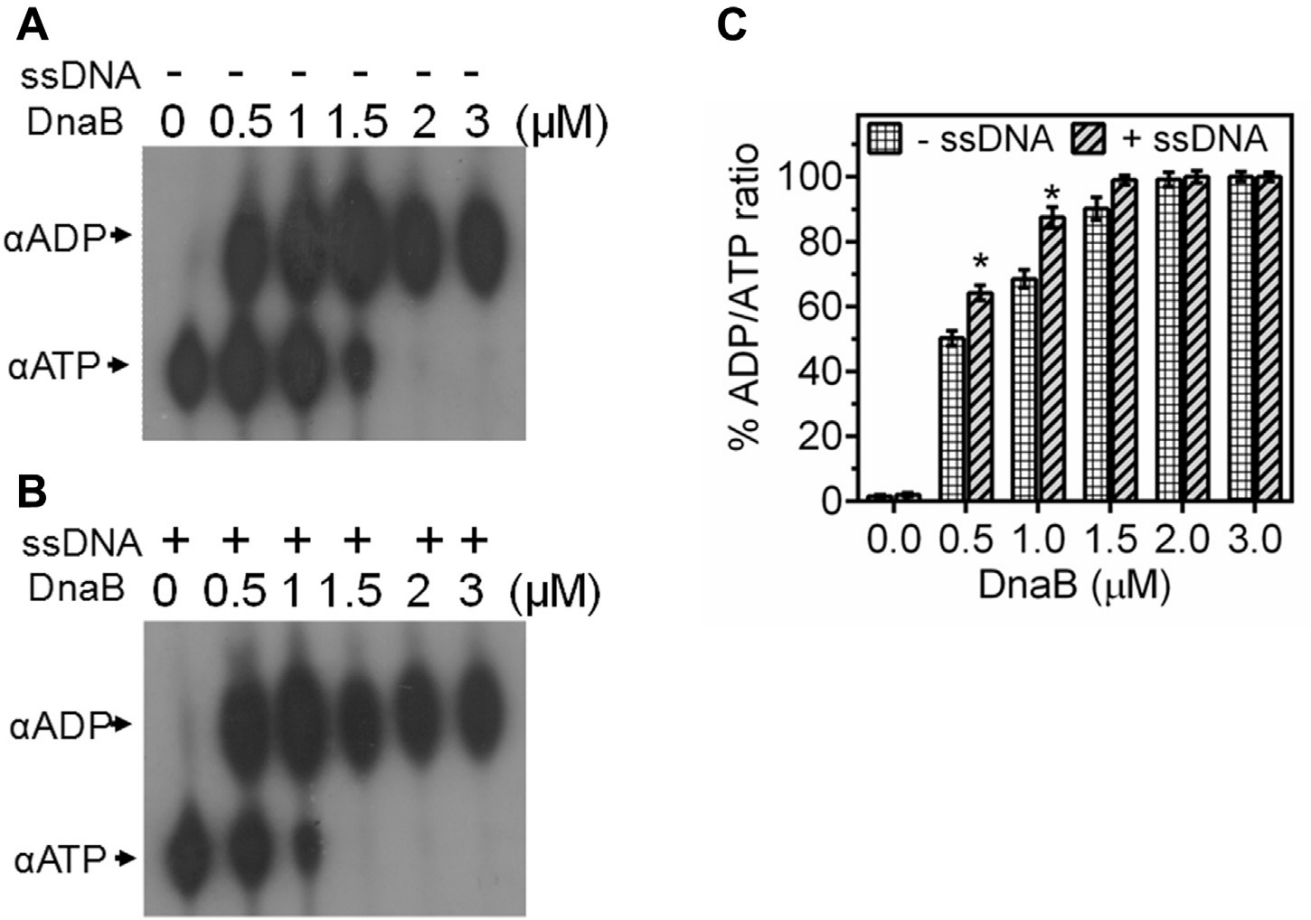
**ATPase activity of purified recombinant drDnaB.** An increasing concentration of drDnaB (DnaB) was incubated with [^32^P]-αATP (αATP) in the absence (*A*) and presence (*B*) of ssDNA and generation of [^32^P]-αADP (αADP) product was detected on TLC. Spot intensity was quantified densitometrically and the percent of ADP/ATP ratios wasplotted as mean ± SD (n = 3) (*C*). Results were analyzed using Student’s *t*-test and a significant difference in data set with *p* values of 0.05 or less is marked with (∗).

Binding of recombinant drDnaB to the *oriCI* was checked by EMSA. drDnaB showed nonspecific binding to *oriCI* as chase with nonspecific dsDNA significantly competed out *oriCI* bound to drDnaB. The affinity of drDnaB for *oriCI* (Kd = 8.89 ± 0.92 μM) did not change in the presence of ATP (Kd = 8.23 ± 0.41 μM). Interestingly, drDnaB showed approximately threefold higher affinity to ssDNA substrate (Kd = 3.11 ± 0.11 μM) as compared with dsDNA (*oriCI*) (Kd = 8.89 ± 0.92 μM). Further, binding of drDnaB to ssDNA increased by nearly twofold in the presence of ATP (Kd = 1.42 ± 0.05 μM) (Fig. 6) and approximately threefold in the presence of ATPγS (1.02 ± 0.06 μM) (Fig. S6). Addition of ATP increased drDnaB affinity for ssDNA but not for dsDNA. These results suggested that drDnaB preferentially binds to ssDNA over dsDNA both in the presence and absence of ATP. Similar to drDnaA, drDnaB could hydrolyze [^32^P]-αATP to [^32^P]-αADP (Fig. 7*A*). The stimulatory effect of ssDNA on ATP hydrolysis was observed *albeit* at a very low level and only at the lower ATP to protein ratios (Fig. 7, *B* and *C*). Similar observations were reported earlier for other DnaB homologs (40, 41). These results suggested that drDnaB is an ssDNA-binding ATPase and its binding to ssDNA is affected by ATP hydrolysis *in vitro*.

**Figure 8:**
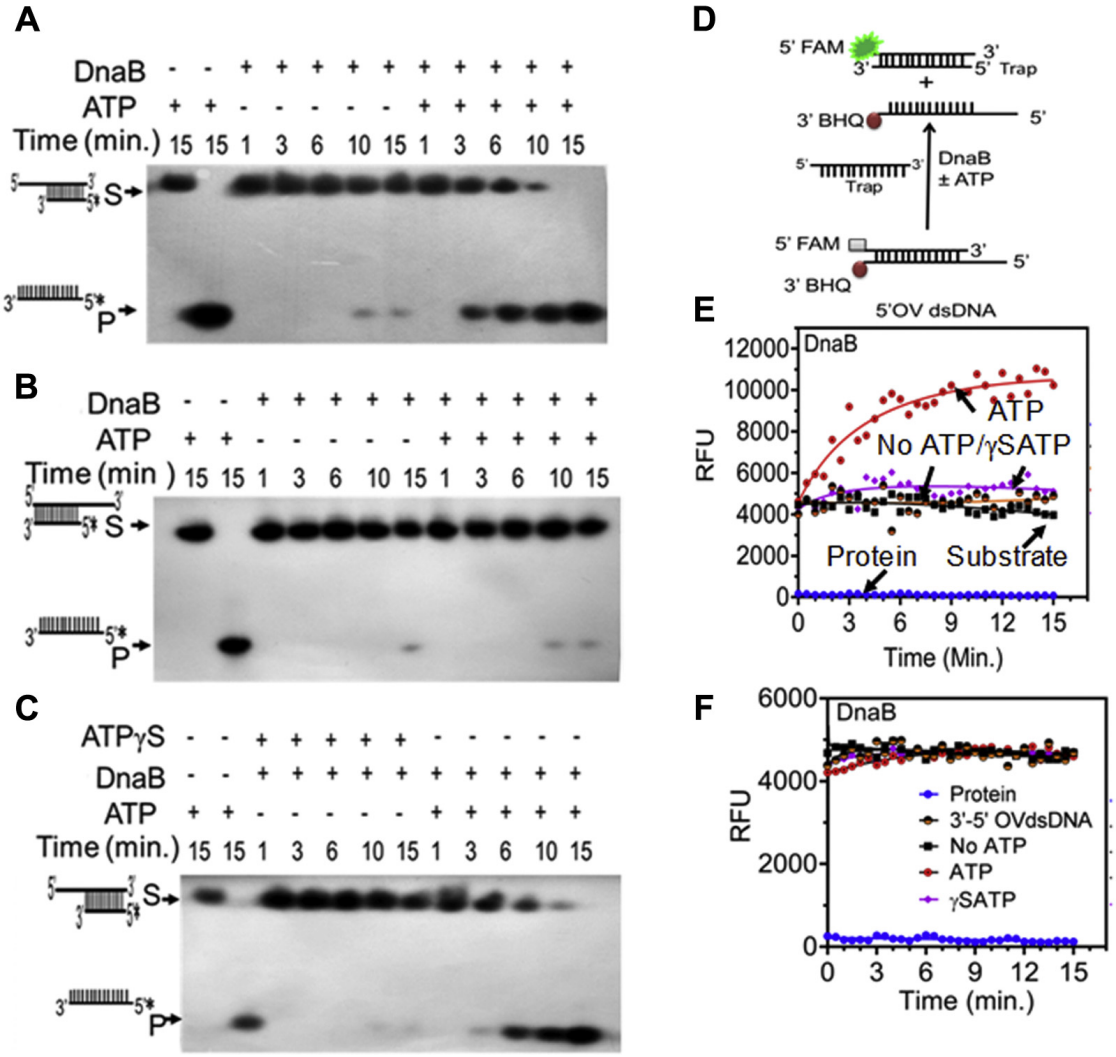
**Helicase activity assay of recombinant drDnaB.** The purified recombinant drDnaB (DnaB) (2 μM) was incubated with radiolabeled dsDNA having 5’ overhang (*A*) and 3’ overhang (*B*) in the presence and absence of ATP. The role of ATP hydrolysis on helicase activity was monitored with dsDNA with 5’ overhang in the presence and absence of ATP and ATP-γ-S for different time points (*C*). Products were analyzed on denaturing PAGE and autoradiograms were developed. The helicase activity was also monitored using FRET assay on a substrate containing FAM at 5’ end and BHQ at 3’ end of dsDNA (*D*). Loss of FRET was monitored as a gain of FAM emission with the substrate having 5’ overhang (*E*) and 3’ overhang (*F*) as a function of time in the presence and absence of ATP and ATPγS. Data represent the sets of the reproducible experiment repeated three times.

DnaBs are characterized as replicative helicases and play an essential role in *oriC*-mediated DNA replication in other bacteria (42, 43, 44). Therefore, the helicase activity of recombinant drDnaB was checked on dsDNA having either 3′ or 5′ overhangs and blunt end as a substrate in the presence and absence of ATP. The subsequent products were monitored on the gel as well as using a Fluorescence Resonance Energy Transfer (FRET)-based assay. Results showed that drDnaB is able to unwind only dsDNA with 5′ overhang, not with 3′ overhang (Compare Fig. 8*A* with Fig. 8*B*) or blunt end substrate (Fig. S7). This 5′ → 3′ dsDNA helicase activity required the hydrolyzable ATP as no DNA unwinding was observed when ATP was substituted with ATPγS (Fig. 8*C*). Similar results were obtained in FRET assays with FAM as a donor and BHQ as an acceptor moiety (Fig. 8*D*). Results showed an increased fluorescence in the presence of drDnaB and ATP, which was lost when ATPγS was substituted for ATP (Fig. 8, *E* and *F*). Interestingly, drDnaB showed binding to blunt end dsDNA substrate, even though no helicase activity was observed for the same. These results suggested that drDnaB is an ATP-dependent 5′ → 3′ dsDNA helicase, which is consistent with the observed characteristics of known DnaB helicases from other bacteria (42, 45, 46).

### drDnaA and drDnaB show homotypic and heterotypic interactions

**Figure 9:**
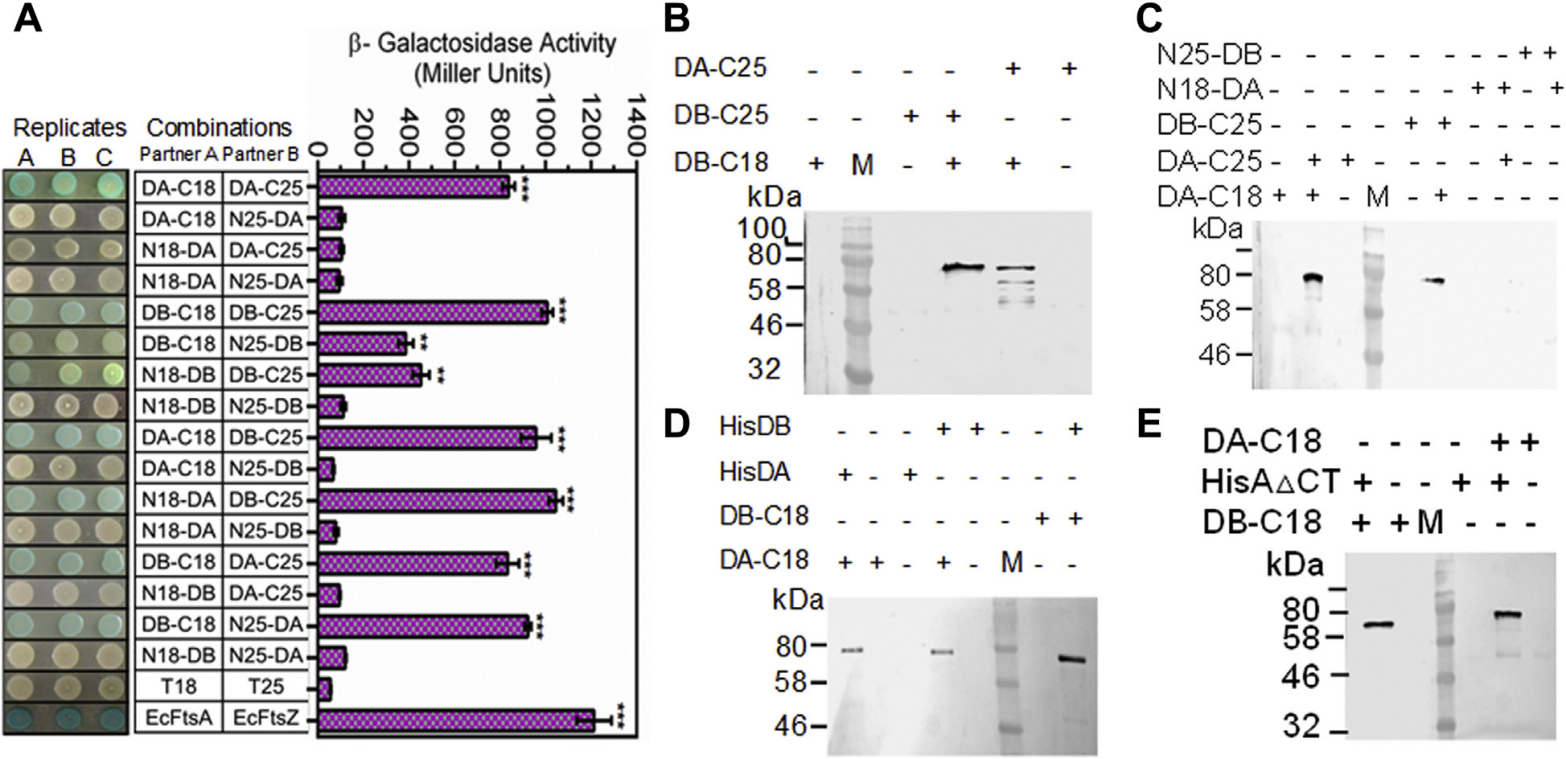
**Interaction between drDnaA and drDnaB proteins.** The translation fusions of drDnaA and drDnaB with T18/T25 tags at N terminal (N18/N25-DA and N18/N25-DB) and C terminal (DA-C18/C25 and DB-C18/C25) were expressed in *E. coli* BTH 101 cells in different combination. The expression of β-galactosidase was checked in the spot test and liquid culture (*A*). Total proteins from *E. coli* expressing some of these combinations were immunoprecipitated with T25 antibodies and interacting partners were detected using T18 antibodies (*B* and *C*). The pUT18 (T18) and pKNT25 (T25) vectors were used as negative control while *E. coli* FtsA (EcFtsA) and FtsZ (EcFtsZ) were used a positive control. *In vivo* interaction of drDnaA (DA-C18), DnaA with histidine tag (HisDA), drDnaB (DB-C18), drDnaB with histidine tag (HisDB) (*D*), and C-terminal truncated drDnaA with histidine tag (His-AΔCT) (*E*) was monitored in *D. radiodurans* R1 (WT) expressing these recombinant proteins in different combinations on the plasmids. Total proteins were precipitated with polyhistidine antibodies and perspective interacting partners were detected using antibodies against the T18 domain of CyaA. Data given in *A* panel was analyzed by Student’s *t*-test and *p* values less than 0.01 and 0.001 were denoted as (∗∗) and (∗∗∗), respectively.

The possible interaction between drDnaA and drDnaB proteins was monitored using bacterial two-hybrid (BACTH) system and co-immunoprecipitation. Both N-terminal and C-terminal fusions of drDnaA and drDnaB with T18 and T25 tags of BACTH were coexpressed in *E. coli* BTH101 (*cyaA*^−^) in different combinations (Fig. S8). The expression of β-galactosidase upon reconstitution of an active CyaA from its T18 and T25 domains that would indicate that the interactions of target proteins were monitored. The drDnaA and drDnaB proteins tagged with T18 and T25 through their C terminals showed both homotypic and heterotypic interactions in *E. coli* (Fig. 9*A*). Some of these interactions were further confirmed by immunoprecipitation and results agreed with the BACTH analysis in *E. coli* (Fig. 9, *B* and *C*). *In vivo* interaction of these proteins was monitored in *D. radiodurans*. For this the whole cell extracts from the cells coexpressing drDnaA or drDnaB with T18 tag and/or polyhistidine tag on the respective plasmids were immunoprecipitated using polyhistidine antibodies, and the potential interacting partner was detected using anti-T18 antibodies. Results confirmed both homotypic and heterotypic interactions of these proteins *in vivo* (Fig. 9*D*). Further, the DnaAΔCt protein also showed interaction with full-length drDnaA and drDnaB (Fig. 9*E*) indicating that CTD is not required for the interaction. Since, C-terminal domain of drDnaA is required for *oriCI* binding but not for both homotypic and heterotypic interaction with drDnaA and drDnaB, the interaction of these proteins seems to be independent of drDnaA interaction with *oriCI*. These results suggested that both these proteins interact through their N-terminal domains as reported earlier for the known homologs (1, 47, 48, 49). Further studies would require for the exact mapping of the interaction domains or residues of these proteins.

### PprA modulates *in vitro* function of drDnaA but not drDnaB

**Figure 10:**
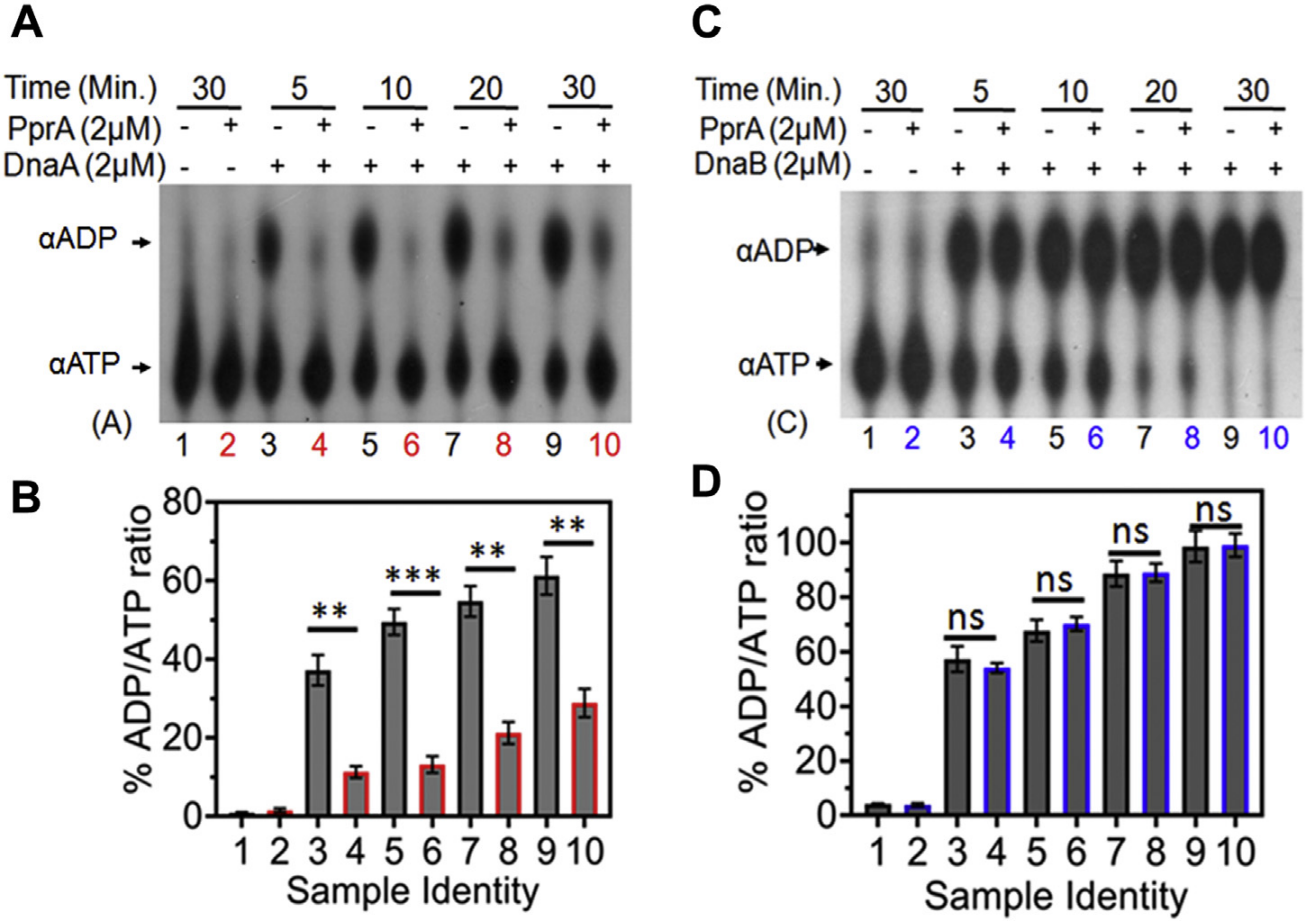
**Effect of recombinant PprA on ATPase activity of recombinant drDnaA and drDnaB.** Recombinant PprA was incubated with drDnaA (DnaA) (*A* and *B*) and drDnaB (DnaB) (*C* and *D*) separately. ATP hydrolysis was monitored using [^32^P]-αATP (αATP) at different time interval and generation of [^32^P]-αADP (αADP) product was estimated. Percentage of ADP to ATP ratios was plotted as the mean ± SD (n = 3) (*B* and *D*). Data sets were analyzed by Student’s *t*-test and the significant difference if any is given as the *p* values less than 0.01 and 0.001 were denoted as (∗∗) and (∗∗∗), respectively and nonsignificance is marked as (ns).

The ATP hydrolysis is the key feature of bacterial DnaA and DnaB proteins, and this activity is required for the initiation and progression of *oriC* mediated replication. The recombinant drDnaA and drDnaB showed ATPase activity *in vitro* (Figs. 4 and 7). The effect of histidine-tagged PprA (His-PprA) on the ATPase activity of both drDnaA and drDnaB was checked *in vitro*. The ATPase activity of drDnaA was significantly reduced in the presence of an equimolar concentration of His-PprA irrespective of the order of addition of these proteins in the reaction mixture (Fig. 10, *A* and *B*). There was no effect of His-PprA on either ATPase activity (Fig. 10, *C* and *D*) or 5′→3′ dsDNA helicase activity (Fig. S9) of drDnaB *in vitro*. His-PprA did not affect drDnaB function *in vitro* and also did not hydrolyze ATP into ADP by itself. Therefore, the possibility of ATP degradation or sequestration by His-PprA affecting the ATPase activity of drDnaA was ruled out. The effect of histidine tag on PprA function was further evaluated. *In trans* expression of His-PprA showed nearly complete complementation to the loss of gamma radioresistance in *pprA* mutant (Fig. S10*A*). These results suggested that PprA affects drDnaA functions. Since the ATPase activity of DnaA is required for *oriC*-dependent replication initiation in bacteria (1, 6), the suppression of ATPase activity of drDnaA by PprA may be an important step in *oriCI* regulation in *D. radiodurans*.

### PprA negatively regulates drDnaA and drDnaB interaction in surrogate *E. coli*

**Figure 11:**
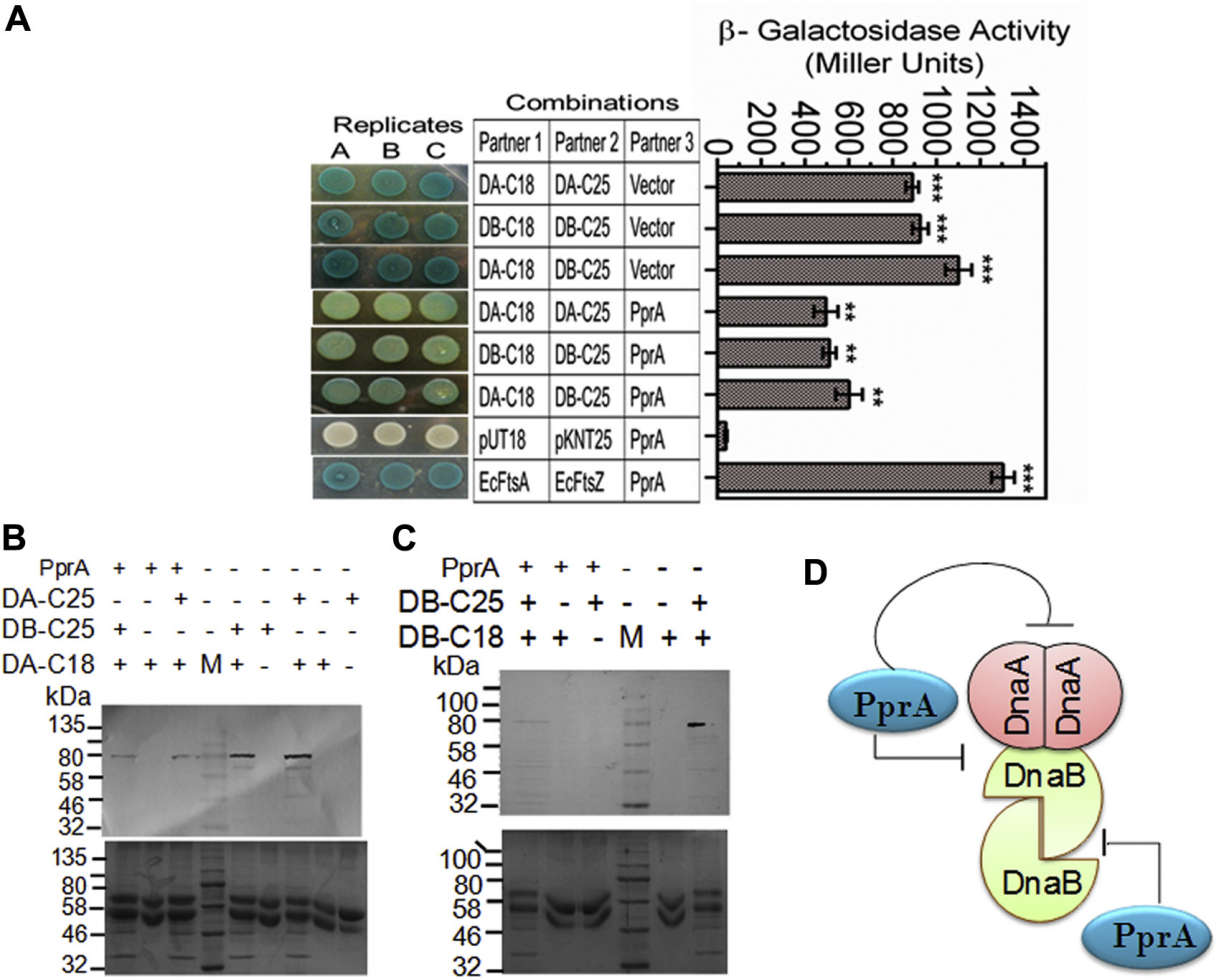
**Influence of PprA on drDnaA and drDnaB interaction in surrogate *E.coli*.***E. coli* BTH 101 cells coexpressing C-terminal fusions of T18/T25 tags with drDnaA (DA-C18, DA-C25), DnaB (DB-C25, DB-C18), and PprA in different combinations. The expression of β-galactosidase as an indication of two proteins interaction was monitored in the spot test and liquid assay (*A*). The pUT18 and pKNT25 vectors were used as negative control while *E. coli* FtsA (EcFtsA) and FtsZ (EcFtsZ) were used a positive control. Total proteins of *E. coli* expressing some of these combinations were immunoprecipitated using T25 antibodies and interacting partners were detected by T18 antibodies (*B* and *C*). In *B* and *C*, the *upper panels* are immunoblots while *lower panels* are corresponding SDS-PAGE gel. PprA's influence on the interaction of these proteins is summarized (*D*).

Since PprA showed interaction with drDnaA or drDnaB with different affinities, the possibility of PprA affecting drDnaA and drDnaB interactions was tested using bacterial two-hybrid system. The *E. coli* BTH101 cells coexpressing drDnaA and drDnaB in different combination were transformed with PprA expressing plasmid (pSpecpprA) (50) and the expression of β-galactosidase activity was monitored. We observed that the expression of β-galactosidase activity originating from the established interaction of drDnaA and drDnaB (Fig. 9) was reduced in the presence of PprA as compared with the control with no PprA (Fig. 11, *A*–*C*). This effect of PprA was observed on homotypic and heterotypic interactions of both the replication proteins (Fig. 11*D*). These results suggested that PprA interferes with the oligomerization of drDnaA and drDnaB proteins of *D. radiodurans*.

## Discussion

Molecular basis of regulation of DNA replication initiation has been extensively studied in bacteria with limited copies of the single circular chromosome. A significant proportion of bacteria harbor multiple genome elements, which are split into two or more chromosomes. Though many of the important pathogenic and plant symbiotic bacteria (*e.g.*, *Burkholderia*, *Brucella*, *Vibrio*, *Shinorhizobium*, etc.) and *D. radiodurans* harbor multipartite genomes, most of our current knowledge on the DNA replication of multipartite genomes has come from the study on *V. Cholerae*. In *V. cholerae*, Chr I replication is initiated by DnaA protein while Chr II replication is initiated by RctB protein that binds and unwinds an array of repeats present in *oriCII* of Chr II (10, 12, 13, 51). Since Chr I is larger than Chr II in *V. cholerae*, the temporal regulation of replication initiation could help in synchronization of replication termination of both the chromosomes before the onset of cell division (52, 53, 54).

*D. radiodurans* harbors a polyploid multipartite genome comprising two chromosomes and two plasmids (20). Molecular mechanism responsible for control of DNA replication initiation in this bacterium has not been reported yet. Homology search for the known replication initiator proteins revealed the presence of *dnaA* and *dnaB* genes, encoding for putative replication initiator proteins drDnaA and drDnaB, on the chromosome I of this bacterium. However, there was no homology with the known replication initiation proteins encoded on secondary genome elements (*e.g.*, RctB and RepA) of other bacteria, in the genome of this bacterium (data not shown). PprA (DR_A0346), a pleiotropic protein of *D. radiodurans*, has been earlier characterized for its role in radioresistance. Here, we have brought forth some evidence to suggest that PprA regulates ploidy of Chromosome I and II and inhibits the activity of DnaA, the initiator protein, by direct interaction in *D. radiodurans*. *In silico* sequence analysis identified putative *oriC*-like sequence in chromosome I (*oriCI*) comprising two AT-rich clusters (12 bp and 20 bp), two GATC regions, and 13 AT-rich DnaA boxes with consensus sequence similar to that of *E. coli* (eight perfect DnaA boxes) or *Cyanothece* 51142, *T. thermophilus*, and *B. subtilis* (5 imperfect DnaA boxes) (Fig. S1). The *oriC1* was located between *drdnaA* and *drdnaN* genes.

Characterization of the purified recombinant proteins using *in vitro* studies showed that both drDnaA and drDnaB have ATPase activity. Similar to other bacteria (32, 55, 56, 57, 58), drDnaA showed sequence-specific interaction with *oriCI*, specifically with a higher affinity for the *E. coli*-like DnaA boxes as well as in the presence of ATP suggesting its probable role as the replication initiator protein. drDnaB binds DNA in a nonspecific manner with a preference of ssDNA over dsDNA. The ATPase activity of drDnaA and drDnaB was stimulated by *oriCI* and ssDNA, respectively. The DnaBs or its homologs from different bacteria have shown different polarities in dsDNA helicase functions (59, 60). Similar to DnaB helicases from *E. coli* (42), *Helicobacter pylori* (46), *Mycobacterium tuberculosis* (41), and in *Bacillus anthracis* (60), drDnaB showed ATP dependent 5′ → 3′ helicase activity on dsDNA with 5’-overhangs.

The *oriC*-mediated initiation of DNA replication requires a minimum of DnaA binding at *oriC* site, the recruitment of (DnaB)_6_-(DnaC)_6_ hexameric replicative helicase, DNA unwinding by helicase function, and energy-dependent elongation of replication fork (6, 43, 61). We observed that drDnaA and drDnaB undergo both homotypic and heterotypic oligomerization and interact with *oriCI* region and ssDNA, respectively. It is known that energy from the ATP hydrolysis by DnaA is used for melting of the AT-rich region in *oriC* necessary for the initiation of replication (1, 6). The presence of ATPase activity in drDnaA and drDnaB proteins and its stimulation by *oriCI* and ssDNA, respectively, as well as ATP-dependent dsDNA helicase function in drDnaB are therefore some of the typical characteristics known for the similar proteins in other bacteria. Altogether, above findings suggest that drDnaA and drDnaB protein confer the desired function to constitute the replication initiation complex with *oriCI* in *D. radiodurans*. Characterization of *oriCI* and replication initiation proteins indicated a replication initiation machinery similar to that present in *E. coli*. The loss of DNA replication initiation regulatory mechanisms would result in a relaxed chromosome copy number phenotype in the cell. Our results demonstrated an increase in the genomic content and ploidy levels in *D. radiodurans* cells deficient in PprA indicating a relaxed replication initiation control as compared with the wild-type cells (Fig. 1). We next asked if PprA modulates the activity of the initiation proteins. Our results showed that PprA inhibited the homotypic and heterotypic macromolecular interactions between drDnaA and drDnaB proteins. In addition, PprA specifically inhibited the ATPase activity of drDnaA while having no effect on the ATPase and dsDNA helicase activity of drDnaB. These results suggest that PprA might regulate the replication initiation by inhibiting drDnaA function and oligomerization of drDnaA and drDnaB. Nonetheless, a direct effect on the replication initiation in PprA mutant remains to be seen and a conditional knockout of the genes encoding these proteins will confirm these functions *in vivo*.

Regulation of DNA replication by a cell cycle regulatory protein competing for binding to *oriC* site was suggested earlier (62); however, absence of the specific interaction of PprA with *oriCI* has ruled out this possibility (Fig. S10, *B* and *C*). Interestingly, treatment with Hydroxy Urea (HU), a replication inhibitor, improved post irradiation recovery (PIR) of wild-type *D. radiodurans* (Fig. S11). It may be possible that the delayed PIR in the absence of PprA might be due to the loss of replication initiation control, which is otherwise necessary for rapid PIR in *D. radiodurans*. Different mechanisms have been reported for the regulation of DNA replication in different bacteria. In *E. coli* the initiation of DNA replication is regulated by the differential affinity of DnaA and SeqA proteins to methylated and hemimethylated *oriC*, respectively, and by the regulated inactivation of DnaA by Hda (5). Further, CtrA involvement in the regulation of DNA replication in *Caulobacter crescentus* (63) and *CrtS* protein interaction with methylated DNA in the case of *V. cholerae* (15) have been reported. Involvement of genome segregation proteins such as Soj and Spo0J in case of *B. subtilis* (64, 65, 66) and ParA and ParB in the regulation of secondary genome copy number in *D. radiodurans* (67, 68) have also been suggested.

In conclusion, we have characterized *oriCI*, drDnaA, and drDnaB, the components involved in *oriC*-mediated initiation of bacterial chromosome replication in multipartite genome harboring radioresistant bacterium *D. radiodurans*. PprA, a protein with pleiotropic functions in this bacterium, was found to regulate genome ploidy and the physicochemical properties of drDnaA and drDnaB necessary for their functions in chromosome replication. Further studies would be required to address if PprA acts as a checkpoint regulator of *oriC*-mediated DNA replication under normal conditions or during DNA double-strand break (DSB) repair. Also, if the phosphorylation status of PprA (50) has any role in these functions would be worth investigating independently.

## Experimental procedures

### Bacterial strains and plasmids

All the bacterial strains, plasmids, and oligonucleotides used in this study are given in Tables S1 and S2, respectively. *D. radiodurans* R1 (ATCC13939) was grown in TGY (tryptone (1%), glucose (0.1%), and yeast extract (0.5%)) medium at 32 °C (69). *E. coli* strain NovaBlue was used for cloning and maintenance of all the plasmids while *E. coli* strain BL21(DE3) pLysS was used for the expression of recombinant proteins. *E. coli* strain BTH 101 was used for Bacterial Two-Hybrid System (BACTH)-based protein–protein interaction studies. Standard protocols for all recombinant techniques were used as described in (70). Molecular biology-grade chemicals and enzymes were procured from Merck Inc and New England Biolabs. Radiolabeled nucleotides were purchased from the Department of Atomic Energy—Board of Radiation and Isotope Technology (DAE-BRIT), India.

### Cloning, expression, and purification of proteins

The drDnaA was expressed from pETDnaA as described in (71). The dr*dnaB* (DR_0549) gene was PCR amplified from the genomic DNA of *D. radiodurans* R1 using pETdnaBF and pETdnaBR primers (Table S2). The PCR product was cloned in pET28a(+) plasmid at *Bam*HI and *Eco*RI sites to yield pETDnaB plasmid (Table S1). The C-terminal (domain IV) truncation of drDnaA was made using pETDAΔCtR primer along with pETdnaAF (Table S2), and the resulting plasmid was named pETDAΔCt (Table S1). The recombinant plasmids were sequenced, and the correctness of inserts was ascertained. The recombinant proteins were purified by nickel affinity chromatography as described earlier (65). Fractions showing more than 95% purity were pooled and dialyzed in buffer A containing 200 mM NaCl and further purified from HiTrap Heparin HP affinity columns (GE Healthcare Life sciences) using a linear gradient of NaCl. Different fractions were analyzed on SDS-PAGE and fractions containing desired protein were pooled and precipitated with 30% w/v ammonium sulphate at 8 °C. The precipitate was dissolved in R-buffer (20 mM Tris-HCl pH 7.6, 0.1 mM EDTA, 0.5 mM DTT) containing 1 M NaCl. After centrifugation at 16,000 rpm for 30 min, the supernatant containing soluble proteins was processed for gel filtration chromatography.The purified protein was dialyzed in dialysis buffer containing 20 mM Tris-HCl pH 7.6, 200 mM NaCl, 50% glycerol, 1 mM MgCl_2,_ 0.5 mM DTT, and 1 mM PMSF. Similarly, PprA was expressed from pETpprA and purified as described in (72) followed by gel filtration as described above. The correct refolding of all purified proteins was confirmed by circular dichroism (CD) spectroscopy in phosphate buffer as described previously (67) (Fig. S1, *B*, *D*, *F* and *H*).

### Protein–DNA interaction studies

The interaction of recombinant proteins with different forms of DNA was studied using EMSA as described earlier (67, 71). In brief, the 500 bp *oriCI* region (1272–1771) containing 13 repeats of 9 mer of DnaA boxes (T(A/G)TA(T)TCCACA) was PCR amplified using OriIFw and OriIRw primers (Table S1 and Fig. S2) and gel purified. In addition, different fragments of *oriCI* with 11, 7, 3, and 1 repeat(s) were also either PCR amplified or chemically synthesized and annealed (Fig. S3). DNA substrates were labeled with [γ-^32^P] ATP using T4 polynucleotide kinase. Approximately 30 nM labeled substrate was incubated with different concentrations of recombinant drDnaA in a reaction buffer B containing 50 mM Tris-HCl (pH 8.0), 75 mM KCl, 5 mM MgSO_4_, and 0.1 mM DTT at 37 °C for 15 min. The reaction was performed in the presence and absence of 1 mM ATP. For the competition assay, a saturating concentration of protein was incubated with radiolabeled DNA substrate before the addition of different concentrations of nonspecific competitor cold DNA of similar length (regions of *ftsZ* gene in Table S1) and further incubated as per experimental requirements. Similarly, oligonucleotide 99F (73) was radiolabeled at 5’ end and used as an ssDNA substrate for interaction with drDnaB. The interaction of drDnaB with *oriCI* (dsDNA) was monitored in the absence and presence of 1 mM ATP as described for drDnaA above. The reaction mixtures were separated on 6 to 8% native PAGE gels, the gels were dried, and autoradiograms were developed on X-ray films. The band intensity of unbound and bound fraction was quantified densitometrically and computed using Image J 2.0 software. The fraction of DNA bound to the protein was plotted against the protein concentrations by using GraphPad Prism6, and the Kd values for the curve fitting of individual plots were determined as described in (67).

### DNA helicase activity assay

The unwinding of dsDNA substrates (with 5’ overhang as well as 3’ overhang) by drDnaB (2 μM) in the presence of different combinations of 1 mM ATP and 2 μM PprA was monitored using FRET approach in 96-well plates as described in (74). In brief, the substrates used were FAM and BHQ labeled complementary oligonucleotides (Table S1). For the preparation of overhang dsDNA, the oligonucleotides of complementary strands producing 5’overhang or 3’ overhang were mixed, heated at 95 °C for 10 min, and then slowly annealed by switching off the heating block for overnight. The helicase assays were performed in volume of 100 μl in helicase buffer (25 mM Tris pH 7.6, 10 mM NaCl, 25 mM KCl, 1 mM MgCl_2,_ 100 μM DTT, 100 μg/ml BSA, and 2% glycerol) at 37 °C. To avoid reannealing of unwound DNA, excess of DNA trap complementary to the BHQ labeled DNA was added to each reaction mixture. The reaction was excited at 490 nm and emission was recorded at 520 nm. The fluorescence signals were monitored at an interval of 30 s using a microplate reader (Biotek Synergy H1).

Similarly, gel-based DNA helicase activity assay of drDnaB was carried out as described in (74). In brief, the 3′ overhang substrate was made by annealing the [^32^P] radiolabeled 3OV99 F (5′ TTTTTGCGGTTCATATGGAATTCC3′) with 99F (5′GGAATTCCAATGAACCGCAAAACCGCAAAAACCGTACCGA3′) as described in (74). Similarly, a 5′ overhang substrate was generated by annealing of radiolabeled 5OV99F (5′TCGGTACGGTTTTTGCGGTTCATA3′) with 99F oligonucleotides. The annealed substrates were incubated with 2 μM drDnaB with or without 2 μM PprA proteins in the presence and absence of 1 mM ATP or ATP-γS in helicase assay buffer at 37 °C. Aliquots were taken at different time intervals and reactions were stopped with stop solution (50 mM KCl, 20% glycerol, 20 mM EDTA, 125 μg Proteinase K, 0.2% SDS) at 37 °C for 10 min. The products were separated on 8% denaturing PAGE, dried, and exposed to X-ray films. The autoradiograms were developed and documented.

### Bacterial two-hybrid (BACTH) system assay

drDnaA and drDnaB interactions were monitored in the absence and presence of PprA using a BACTH, described in (75, 76, 77, 78). In brief, the coding sequence of DR_0002 (drDnaA) was cloned at *Bam*HI and *Eco*RI sites in pUT18, pUT18C, and pKT25 plasmids to yield pUT18DA, pUT18CDA, and pKTDA, respectively. Similarly, coding sequences of DR_0549 (drDnaB) were cloned at *Kpn*I and *Eco*RI sites in pUT18, pUT18C, and pKT25 plasmids to yield pUT18DB, pUT18CDB, and pKTDB, respectively. The construction of pKNTDA and pKNTDB plasmids has been described in (68) and pUTpprA in (23). *E. coli* strain BTH101 (*cyaA*^−^) was cotransformed with these plasmids in different combinations and expression of β-galactosidase was monitored as described earlier (76). *E. coli* BTH101 harboring only vectors were used as negative control while those harboring pUTEFA and pKNTEFZ (79) were used as a positive control. For monitoring the effect of PprA on drDnaA and drDnaB interactions, the PprA was expressed on pSpecpprA (50), whereas the p11559 vector was used for negative control. Expression of β-galactosidase as an indication of protein–protein interaction was monitored using spot assay and in liquid culture, and β-galactosidase activity was calculated in Miller units as described in (76).

### Co-immunoprecipitation assay

Protein–protein interactions in surrogate *E. coli* (BTH101) and *D. radiodurans* were monitored by co-immunoprecipitation as described in (76, 77). In brief, the total proteins of the recombinant BTH101 cells coexpressing drDnaA and drDnaB proteins on BACTH plasmids (Table S1) were immunoprecipitated using polyclonal antibodies against T25 and counterpart was detected using T18 monoclonal antibodies raised in the mouse. PprA’s effect on the interactions among drDnaA and drDnaB was studied by *in trans* expression of PprA from pSpecpprA plasmid. Signals were detected using anti-mouse secondary antibodies conjugated with alkaline phosphatase in the presence of BCIP/NBT substrates (Roche Biochemical, Mannheim). Similarly, for monitoring interactions among drDnaA, DnaAΔCt, and drDnaB in *D. radiodurans* by co-immunoprecipitation, the coding sequences of drDnaA, DnaAΔCt, and drDnaB with polyhistidine tag were PCR amplified using pETHisFw and pETHisRw primers and cloned in pRADgro plasmid (80) at *Apa*I and *Xba*I sites to yield pRADhisDA, pRADhisDACt, and pRADhisDB, respectively (Table S1). The expressions from these plasmids were confirmed in *D. radiodurans* using anti-polyhistidine antibodies (Fig. S7, *E* and *F*). In addition, the coding sequences of T18-tagged drDnaA and drDnaB were PCR amplified using BTHF(pv) and BTHR(pv) primers from pUT18DA and pUT18DB plasmids and cloned in p11559 plasmid at *Nde*I and *Xho*I sites to yield pV18DA and pV18DB, respectively (Table S1). Expression of T18-tagged drDnaA and drDnaB from pV18DA and pV18DB in *D. radiodurans* was confirmed using anti-T18 antibodies (Fig. S8*A*). These plasmids were cotransformed in different combinations in *D. radiodurans* and induced with 5 mM IPTG as required. The cell-free extracts of *D. radiodurans* expressing drDnaA, DnaAΔCt, and drDnaB in different combinations were prepared and immunoprecipitated using antipolyhistidine antibodies as described earlier (67). The immunoprecipitates were purified using Protein G Immunoprecipitation Kit (Cat. No. IP50, Sigma-Aldrich Inc) and were separated on SDS-PAGE for western blotting using monoclonal antibodies against T18 as described above. To show the interaction of PprA with drDnaA or drDnaB, the wild-type cells of *D. radiodurans* expressing native PprA were transformed with pV18DA or pV18DB and expression of T18-tagged drDnaA or drDnaB was induced with 5 mM IPTG. The cell-free extracts of these transformants were prepared and immunoprecipitated using Anti-PprA antibodies. The purified immunoprecipitates were processed for western blotting using T18 antibodies as described above.

### Surface plasmon resonance

Interaction of drDnaA and drDnaB with PprA was also investigated using surface plasmon resonance (SPR; Autolab Esprit, Netherland) as described in (77). For this, 20 μM PprA was immobilized on a bare gold sensor chip using EDC-NHS chemistry at 20 °C as described in the user manual, which results in ∼200 response units in running buffer [20 mM Tris (pH 7.6)]. The different concentrations (4–20 μM) of recombinant drDnaA or drDnaB incubated with 1 mM ATP and 1 mM MgCl_2_ were used in the mobile phase and passed over the PprA-bound sensor chip in one channel. Reaction buffer containing 20 mM Tris (pH 7.6), 1 mM ATP, and 1 mM MgCl_2_ was used as buffer control to flow from another channel over immobilized PprA. The response units for each concentration of proteins were recorded and normalized with buffer control. Further, data were processed using the inbuilt Autolab kinetic evaluation software (V5.4) to find dissociation constant and plotted after curve smoothening using GraphPad Prizm6 software.

### Thin layer chromatography for ATPase assay

ATPase activity of drDnaA, DnaAΔCt, and drDnaB was measured as the release of [^32^P] αADP from [^32^P] αATP using Thin Layer Chromatography (TLC) as described earlier (67). In brief, different concentrations (0–3 μM) of drDnaA or drDnaB were mixed with 30 nM [^32^P] αATP in the absence and presence of 0.2 pmol dsDNA or 0.1 pmol ssDNA in 10 μl reaction mixture containing buffer B, respectively. The reaction mixture was incubated at 37 °C for 30 min. For monitoring the effect of PprA on ATPase activity, 2 μM PprA was used with 2 μM drDnaA or 2 μM drDnaB as described above. The reaction mixture was stopped at the different time points (0–30 min) with 10 mM EDTA solution and 1 μl of it was spotted on the PEI-Cellulose F^+^ TLC sheet. The spots were air-dried, and products were separated on a solid support in a buffer system containing 0.75 M KH_2_PO_4_/H_3_PO_4_ (pH 3.5). After separation, the TLC sheets were air-dried and exposed to X-ray film. The autoradiograms were developed. Spot intensities of samples were determined by densitometry using Image J 2.0 software. The percentage ratio of ADP to ATP was calculated and plotted using the GraphPad Prizm6 software.

### Fluorescence microscopy

The *D. radiodurans* R1 (WT) and its Δ*pprA* mutant cells were grown till the exponential phase, and the equal number of cells were incubated with 2.5 μg 4’,6-diamidino-2-phenylindole dihydrochloride (DAPI) per ∼10^8^ cells for nucleoid staining. These cells were washed twice in phosphate buffer saline (PBS) and then mounted on slides coated with 0.8% agarose. Cells were imaged in DIC channel (automatic exposure) and DAPI channel (50 milliseconds exposure) under identical conditions for both WT and Δ*pprA* mutant using an Olympus IX83 inverted fluorescence microscope equipped with an Olympus DP80 CCD monochrome camera. Many cells (∼150) from both WT and Δ*pprA* mutant were processed and the fluorescence intensity of DAPI stained nucleoid was measured using the “intensity profile” tool in cellSens1.16 software installed with the microscope. Mean fluorescence intensity was plotted against sample type using GraphPad Prizm6 software. In addition, relative frequency distribution (%) was also plotted against fluorescence intensity using GraphPad Prizm6 software.

### Determination of ploidy in wild type and ΔpprA mutant

The amount of DNA and copy number of all four genome elements such as chromosome I, chromosome II, megaplasmid, and plasmid per cell of wild type and Δ*pprA* mutant were determined as described earlier (67). In brief, the exponentially growing wild type and Δ*pprA* cells were adjusted at an optical density of 600 nm and their cell numbers were determined using a Neubauer cell counter. The collected cells were washed in PBS followed by 70% ethanol wash and lysed in a solution containing 10 mM Tris pH 7.6, 1 mM EDTA, 4 mg/ml lysozyme. The cells were incubated at 37 °C and cellular debris was removed by centrifugation at 10,000 rpm for 5 min. The integrity of isolated genomic DNA was confirmed by agarose gel electrophoresis using 0.8% agarose gel. The DNA content was measured at OD260 nm and the genomic copy number was determined using quantitative real-time PCR as described in (81). For the quantification of genome copy numbers, two different genes showing similar PCR efficiency were taken per replicon. For example, the *ftsZ* and *ftsE* for chromosome I, DR_A0155 and DR_A0002 for chromosome II, DR_B003 and DR_B0076 for megaplasmid, and DR_C001 and DR_C018 for small plasmid (Table S2). PCR efficiency of each gene was analyzed and was found to be >96% for each (data not shown). We have followed the Minimum Information for Publication of Quantitative Real-Time PCR Experiments (MIQE) guidelines (82) for qPCR using Roche Light cycler and the cycle threshold (Cp) values were determined. The experiment was performed using three independent biological replicates for each sample. The Cp value for each gene of the respective replicon was compared with a dilution series of a PCR product of known concentration, as a standard (Fig. S12). The copy number of each replicon by both genes per cell was determined using the cell number present at the time of cell lysis. Thus, an average of copy numbers from two genes per replicon in WT and Δ*pprA* mutant was calculated and represented along with biostatistical analysis.

### Cell survival studies

The *D. radiodurans* cells were grown till early logarithmic phase and divided into two sets. One was treated with 500 mM hydroxyurea for 1 h and other set was kept as control. Both these samples were centrifuged and resuspended into the same volume of fresh TYG medium. Each of these was divided into two aliquots. One aliquot of each set was exposed to 6 kGy gamma radiation and the other was used as SHAM control. These cells were inoculated in normal TYG medium in six replicates and growth was monitored for overnight using Synergy H1 multimode plate reader. Data were analyzed for statistical deviations using GraphPad Prizm6 software and plotted at mean ± SD (n = 6). The wild type, *pprA* mutant, and mutant complemented *in trans* with histidine tagged PprA on a plasmid were exposed to 6 kGy gamma radiation and different dilutions were spotted in TYG agar plate with SHAM control.

## Data availability

All data are contained within the article in form of main figures, table, and supplementary materials.

## Supporting information

This article contains supporting information.

## Conflict of interest

Authors have no financial or nonfinancial competing interests.

## References

[1] Messer W. (2002). The bacterial replication initiator DnaA. DnaA and oriC, the bacterial mode to initiate DNA replication. FEMS Microbiol. Rev..

[2] Mott M.L., Berger J.M. (2007). DNA replication initiation: Mechanisms and regulation in bacteria. Nat. Rev. Microbiol..

[3] Zakrzewska-Czerwińska J., Jakimowicz D., Zawilak-Pawlik A., Messer W. (2007). Regulation of the initiation of chromosomal replication in bacteria. FEMS Microbiol. Rev..

[4] Leonard A.C., Grimwade J.E. (2015). The orisome: Structure and function. Front. Microbiol..

[5] Skarstad K., Katayama T. (2013). Regulating DNA replication in bacteria. Cold Spring Harb. Perspect. Biol..

[6] Chodavarapu S., Kaguni J.M. (2016). Replication initiation in bacteria.

[7] McHenry C.S. (2011). Bacterial replicases and related polymerases. Curr. Opin. Chem. Biol..

[8] Yao N.Y., O’Donnell M. (2010). SnapShot: The replisome. Cell.

[9] Misra H.S., Maurya G.K., Kota S., Charaka V.K. (2018). Maintenance of multipartite genome system and its functional significance in bacteria. J. Genet..

[10] Egan E.S., Fogel M.A., Waldor M.K. (2005). Divided genomes: Negotiating the cell cycle in prokaryotes with multiple chromosomes. Mol. Microbiol..

[11] Egan E.S., Waldor M.K. (2003). Distinct replication requirements for the two *Vibrio cholerae* chromosomes. Cell.

[12] Jha J.K., Baek J.H., Venkova-Canova T., Chattoraj D.K. (2012). Chromosome dynamics in multichromosome bacteria. Biochim. Biophys. Acta.

[13] Ramachandran R., Jha J., Paulsson J., Chattoraj D. (2017). Random versus cell cycle-regulated replication initiation in bacteria: Insights from studying *Vibrio cholerae* chromosome 2. Microbiol. Mol. Biol. Rev..

[14] Koch B., Ma X., Lobner-Olesen A. (2010). Replication of *Vibrio cholerae* chromosome I in *Escherichia coli*: Dependence on dam methylation. J. Bacteriol..

[15] Fournes F., Val M.E., Skovgaard O., Mazel D. (2018). Replicate once per cell cycle: Replication control of secondary chromosomes. Front. Microbiol..

[16] Zahradka K., Slade D., Bailone A., Sommer S., Averbeck D., Petranovic M., Lindner A.B., Radman M. (2006). Reassembly of shattered chromosomes in *Deinococcus radiodurans*. Nature.

[17] Cox M.M., Battista J.R. (2005). *Deinococcus radiodurans* - the consummate survivor. Nat. Rev. Microbiol..

[18] Slade D., Radman M. (2011). Oxidative stress resistance in *Deinococcus radiodurans*. Microbiol. Mol. Biol. Rev..

[19] Misra H.S., Rajpurohit Y.S., Kota S. (2013). Physiological and molecular basis of extreme radioresistance in *Deinococcus radiodurans*. Curr. Sci..

[20] White O., Eisen J.A., Heidelberg J.F., Hickey E.K., Peterson J.D., Dodson R.J., Haft D.H., Gwinn M.L., Nelson W.C., Richardson D.L., Moffat K.S., Qin H., Jiang L., Pamphile W., Crosby M. (1999). Genome sequence of the radioresistant bacterium *Deinococcus radiodurans* R1. Science.

[21] Hansen M.T. (1978). Multiplicity of genome equivalents in the radiation-resistant bacterium *Micrococcus radiodurans*. J. Bacteriol..

[22] Narumi I., Satoh K., Cui S., Funayama T., Kitayama S., Watanabe H. (2004). PprA: A novel protein from *Deinococcus radiodurans* that stimulates DNA ligation. Mol. Microbiol..

[23] Kota S., Charaka V.K., Ringgaard S., Waldor M.K., Misra H.S. (2014). PprA contributes to *Deinococcus radiodurans* resistance to nalidixic acid, genome maintenance after DNA damage and interacts with deinococcal topoisomerases. PLoS One.

[24] Kota S., Rajpurohit Y.S., Charaka V.K., Satoh K., Narumi I., Misra H.S. (2016). DNA gyrase of *Deinococcus radiodurans* is characterized as type II bacterial topoisomerase and its activity is differentially regulated by PprA *in vitro*. Extremophiles.

[25] Adachi M., Hirayama H., Shimizu R., Satoh K., Narumi I., Kuroki R. (2014). Interaction of double-stranded DNA with polymerized PprA protein from *Deinococcus radiodurans*. Protein Sci..

[26] Adachi M., Shimizu R., Shibazaki C., Satoh K., Fujiwara S., Arai S., Narumi I., Kuroki R. (2019). Extended structure of pleiotropic DNA repair-promoting protein PprA from *Deinococcus radiodurans*. FASEB J..

[27] Devigne A., Mersaoui S., Bouthier-de-la-Tour C., Sommer S., Servant P. (2013). The PprA protein is required for accurate cell division of γ-irradiated *Deinococcus radiodurans* bacterium. DNA Repair.

[28] Devigne A., Guérin P., Lisboa J., Quevillon-Cheruel S., Armengaud J., Sommer S., Bouthier de la Tour C., Servant P. (2016). PprA protein is involved in chromosome segregation via its physical and functional interaction with DNA gyrase in irradiated *Deinococcus radiodurans* bacteria. mSphere.

[29] Luo H., Gao F. (2018). DoriC 10.0: An updated database of replication origins in prokaryotic genomes including chromosomes and plasmids. Nucleic Acids Res..

[30] Crooks G.E., Hon G., Chandonia J.M., Brenner S.E. (2004). WebLogo: A sequence logo generator. Genome Res..

[31] Huang H., Song C.C., Yang Z.L., Dong Y., Hu Y.Z., Gao F. (2015). Identification of the replication origins from *cyanothece* ATCC 51142 and their interactions with the DnaA protein: From in silico to *in vitro* studies. Front. Microbiol..

[32] Schaper S., Nardmann J., Lu-der G., Lurz R., Speck C., Messer W. (2000). Identification of the chromosomal replication origin from *Thermus thermophilus* and its interaction with the replication initiator DnaA. J. Mol. Biol..

[33] Moriya S., Fukuoka T., Ogasawara N., Yoshikawa H. (1988). Regulation of initiation of the chromosomal replication by DnaA-boxes in the origin region of the *Bacillus subtilis* chromosome. EMBO J..

[34] Roth A., Messer W. (1995). The DNA binding domain of the initiator protein DnaA. EMBO J..

[35] Sutton M.D., Kaguni J.M. (1997). The *Escherichia coli* dnaA gene: Four functional domains. J. Mol. Biol..

[36] Majka J., Jakimowicz D., Messer W., Schrempf H., Lisowski M., Zakrzewska-Czerwinska J. (1999). Interactions of the *Streptomyces lividans* initiator protein DnaA with its target. Eur. J. Biochem..

[37] Blaesing F., Weigel C., Messer W. (2000). Analysis of the DNA binding domain of *Escherichia coli* DnaA protein. Mol. Microbiol..

[38] Messer W., Blaesing F., Majka J., Nardmann J., Schaper S., Schmidt A., Seitz H., Speck C., Tüngler D., Wegrzyn G., Weigel C., Welzeck M., Zakrzewska-Czerwinska J. (1999). Functional domains of DnaA proteins. Biochimie.

[39] Fujikawa N., Kurumizaka H., Nureki O., Terada T., Shirouzu M., Katayama T., Yokoyama S. (2003). Structural basis of replication origin recognition by the DnaA protein. Nucleic Acids Res..

[40] Biswas E.E., Barnes M.H., Moir D.T., Biswas S.B. (2009). An essential DnaB helicase of *Bacillus anthracis*: Identification, characterization, and mechanism of action. J. Bacteriol..

[41] Zhang H., Zhang Z., Yang J., He Z.G. (2014). Functional characterization of DnaB helicase and its modulation by single-stranded DNA binding protein in *Mycobacterium tuberculosis*. FEBS J..

[42] LeBowitz J.H., McMacken R. (1986). The *Escherichia coli* dnaB replication protein is a DNA helicase. J. Biol. Chem..

[43] Patel S.S., Picha K.M. (2000). Structure and function of hexameric helicases. Annu. Rev. Biochem..

[44] Zawilak A., Cebrat S., Mackiewicz P., Król-Hulewicz A., Jakimowicz D., Messer W., Gosciniak G., Zakrzewska-Czerwinska J. (2001). Identification of a putative chromosomal replication origin from *Helicobacter pylori* and its interaction with the initiator protein DnaA. Nucleic Acids Res..

[45] Soultanas P., Wigley D.B. (2002). Site-directed mutagenesis reveals roles for conserved amino acid residues in the hexameric DNA helicase DnaB from *Bacillus stearothermophilus*. Nucleic Acids Res..

[46] Soni R.K., Mehra P., Choudhury N.R., Mukhopadhyay G., Dhar S.K. (2003). Functional characterization of *Helicobacter pylori* DnaB helicase. Nucleic Acids Res..

[47] Nitharwal R.G., Verma V., Subbarao N., Dasgupta S., Choudhury N.R., Dhar S.K. (2012). DNA binding activity of *Helicobacter pylori* DnaB helicase: The role of the N-terminal domain in modulating DNA binding activities. FEBS J..

[48] Seitz H., Weigel C., Messer W. (2000). The interaction domains of the DnaA and DnaB replication proteins of *Escherichia coli*. Mol. Microbiol..

[49] Matthews L.A., Simmons L.A. (2019). Cryptic protein interactions regulate DNA replication initiation. Mol. Microbiol..

[50] Rajpurohit Y.S., Misra H.S. (2013). Structure-function study of deinococcal serine/threonine protein kinase implicates its kinase activity and DNA repair protein phosphorylation roles in radioresistance of *Deinococcus radiodurans*. Int. J. Biochem. Cell Biol..

[51] Bruhn M., Schindler D., Kemter F.S., Wiley M.R., Chase K., Koroleva G.I., Palacios G., Sozhamannan S., Waldminghaus T. (2018). Functionality of two origins of replication in *Vibrio cholerae* strains with a single chromosome. Front. Microbiol..

[52] Rasmussen T., Jensen R.B., Skovgaard O. (2007). The two chromosomes of *Vibrio cholerae* are initiated at different time points in the cell cycle. EMBO J..

[53] Val M.E., Marbouty M., de Lemos Martins F., Kennedy S.P., Kemble H., Bland M.J., Possoz C., Koszul R., Skovgaard O., Mazel D. (2016). A checkpoint control orchestrates the replication of the two chromosomes of *Vibrio cholerae*. Sci. Adv..

[54] Val M.E., Soler-Bistue A., Bland M.J., Mazel D. (2014). Management of multipartite genomes: The *Vibrio cholerae* model. Curr. Opin. Microbiol..

[55] Yoshikawa H., Wake R.G., Sonenshein A.L., Hoch J.A., Losick R. (1993). Initiation and termination of chromosome replication. *Bacillus Subtilis* and Other Gram-Positive Bacteria.

[56] Zawilak A., Agnieszka K.O.I.S., Konopa G., Smulczyk-Krawczyszyn A., Zakrzewska-Czerwińska J. (2004). *Mycobacterium tuberculosis* DnaA initiator protein: Purification and DNA-binding requirements. Biochem. J..

[57] Calcutt M.J., Schmidt F.J. (1992). Conserved gene arrangement in the origin region of the *Streptomyces coelicolor* chromosome. J. Bacteriol..

[58] Ye F., Renaudin J., Bové J.M., Laigret F. (1994). Cloning and sequencing of the replication origin (oriC) of the *Spiroplasma citri* chromosome and construction of autonomously replicating artificial plasmids. Curr. Microbiol..

[59] Caspi R., Pacek M., Consiglieri G., Helinski D.R., Toukdarian A., Konieczny I. (2001). A broad host range replicon with different requirements for replication initiation in three bacterial species. EMBO J..

[60] Naqvi A., Tinsley E., Khan S.A. (2003). Purification and characterization of the PcrA helicase of *Bacillus anthracis*. J. Bacteriol..

[61] Zawilak-Pawlik A., Nowaczyk M., Zakrzewska-Czerwińska J. (2017). The role of the N-terminal domains of bacterial initiator DnaA in the assembly and regulation of the bacterial replication initiation complex. Genes.

[62] Quon K.C., Yang B., Domian I.J., Shapiro L., Marczynski G.T. (1998). Negative control of bacterial DNA replication by a cell cycle regulatory protein that binds at the chromosome origin. Proc. Natl. Acad. Sci. U. S. A..

[63] Spencer W., Siam R., Ouimet M.-C., Bastedo D.P., Marczynski G.T. (2009). CtrA, a global response regulator, uses a distinct second category of weak DNA binding sites for cell cycle transcription control in *Caulobacter crescentus*. J. Bacteriol..

[64] Ogura Y., Ogasawara N., Harry E.J., Moriya S. (2003). Increasing the ratio of Soj to Spo0J promotes replication initiation in Bacillus subtilis. J. Bacteriol..

[65] Murray H., Errington J. (2008). Dynamic control of the DNA replication initiation protein DnaA by Soj/ParA. Cell.

[66] Scholefield G., Whiting R., Errington J., Murray H. (2011). Spo0J regulates the oligomeric state of Soj to trigger its switch from an activator to an inhibitor of DNA replication initiation. Mol. Microbiol..

[67] Maurya G.K., Kota S., Kumar N.N., Tewari R., Misra H.S. (2019). ParA proteins of secondary genome elements crosstalk and regulate radioresistance through genome copy number reduction in *Deinococcus radiodurans*. Biochem. J..

[68] Maurya G.K., Kota S., Misra H.S. (2019). Characterisation of ParB encoded on multipartite genome in *Deinococcus radiodurans* and their roles in radioresistance. Microbiol. Res..

[69] Schäefer M., Schmitz C., Facius R., Horneck G., Milow B., Funken K.H., Ortner J. (2000). Systematic study of parameters influencing the action of rose bengal with visible light on bacterial cells: Comparison between the biological effect and singlet-oxygen production. Photochem. Photobiol..

[70] Green M.R., Sambrook J. (2012). Molecular Cloning: A Laboratory Manual.

[71] Maurya G.K., Misra H.S. (2020). Characterization of ori and parS-like functions in secondary genome replicons in *Deinococcus radiodurans*. Life Sci. Alliance.

[72] Kota S., Misra H.S. (2006). PprA: A protein implicated in radioresistance of *Deinococcus radiodurans* stimulates catalase activity in *Escherichia coli*. Appl. Microbiol. Biotechnol..

[73] Das A.D., Misra H.S. (2012). DR2417, a hypothetical protein characterized as a novel β-CASP family nuclease in radiation resistant bacterium, Deinococcus radiodurans. Biochim. Biophys. Acta.

[74] Khairnar N.P., Maurya G.K., Pandey N., Das A., Misra H.S. (2019). DrRecQ regulates guanine quadruplex DNA structure dynamics and its impact on radioresistance in *Deinococcus radiodurans*. Mol. Microbiol..

[75] Karimova G., Pidoux J., Ullmann A., Ladant D. (1998). A bacterial two-hybrid system based on a reconstituted signal transduction pathway. Proc. Natl. Acad. Sci. U. S. A..

[76] Maurya G.K., Modi K., Misra H.S. (2016). Divisome and segrosome components of *Deinococcus radiodurans* interact through cell division regulatory proteins. Microbiology (Reading).

[77] Maurya G.K., Modi K., Banerjee M., Chaudhary R., Rajpurohit Y.S., Misra H.S. (2018). Phosphorylation of FtsZ and FtsA by a DNA damage-responsive Ser/Thr protein kinase affects their functional interactions in *Deinococcus radiodurans*. mSphere.

[78] Battesti A., Bouveret E. (2012). The bacterial two-hybrid system based on adenylate cyclase reconstitution in *Escherichia coli*. Methods.

[79] Modi K.M., Misra H.S. (2014). Dr-FtsA, an actin homologue in *Deinococcus radiodurans* differentially affects Dr-FtsZ and Ec-FtsZ functions *in vitro*. PLoS One.

[80] Misra H.S., Khairnar N.P., Kota S., Shrivastava S., Joshi V.P., Apte S.K. (2006). An exonuclease I sensitive DNA repair pathway in *Deinococcus radiodurans*: A major determinant of radiation resistance. Mol. Microbiol..

[81] Breuert S., Allers T., Spohn G., Soppa J. (2006). Regulated polyploidy in halophilic archaea. PLoS One.

[82] Bustin S.A., Benes V., Garson J.A., Hellemans J., Huggett J., Kubista M., Mueller R., Nolan T., Pfaffl M.W., Shipley G.L., Vandesompele J., Wittwer C.T. (2009). The MIQE guidelines: Minimum information for publication of quantitative real-time PCR experiments. Clin. Chem..

