# Supplementary material for: Molecular insights into replication initiation in a multipartite genome harboring bacterium Deinococcus radiodurans
Source: J Biol Chem. 2021 Feb 21;296:100451. doi: 10.1016/j.jbc.2021.100451 (PMC7988490; doi:10.1016/j.jbc.2021.100451)
Supplement: Supplemental Figures S1–S12 and Tables S1–S2 [file mmc1.pdf]

SUPPLEMENTARY MATERIALS

Molecular insights into replication initiation in a multipartite genome harboring bacterium *Deinococcus radiodurans*

Ganesh K. Maurya [$,1,2] , Reema Chaudhary[1,2], Neha Pandey[1,3] and Hari S. Misra *[1,2]

[1] Molecular Biology Division, Bhabha Atomic Research Centre, Mumbai-85

[2] Life Sciences, Homi Bhabha National Institute (DAE deemed to be University) Mumbai-94

[3] Life Sciences, University of Mumbai, Mumbai

[$] Zoology Section, MMV, BHU, Varanasi

* Corresponding authors

Email: hsmisra@barc.gov.in

Supplementary figures - Fig S1, Fig S2, Fig S3, Fig S4, Fig S5, Fig S6, Fig S7, Fig S8, Fig S9, Fig S10 and Fig S11, Fig S12

Supplementary Tables - Table S1 and Table S2

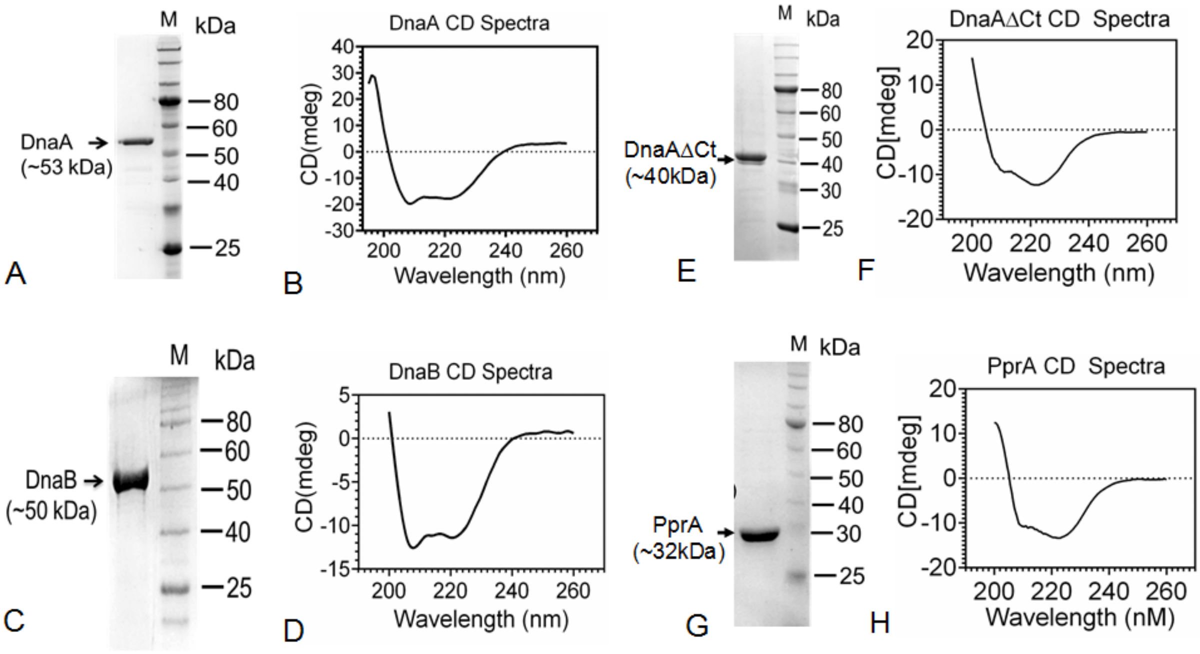

Fig. S1. Secondary structure assessment of purified recombinant proteins used in this study. Recombinant drDnaA (A, B), drDnaB (C, D), DnaAΔCt (E, F) and PprA (G,H) were purified from transgenic *E. coli*. These proteins were analysed on SDS-PAGE (A,C,E,G) and by Circular Dichroism analysis (B,D,F,H).

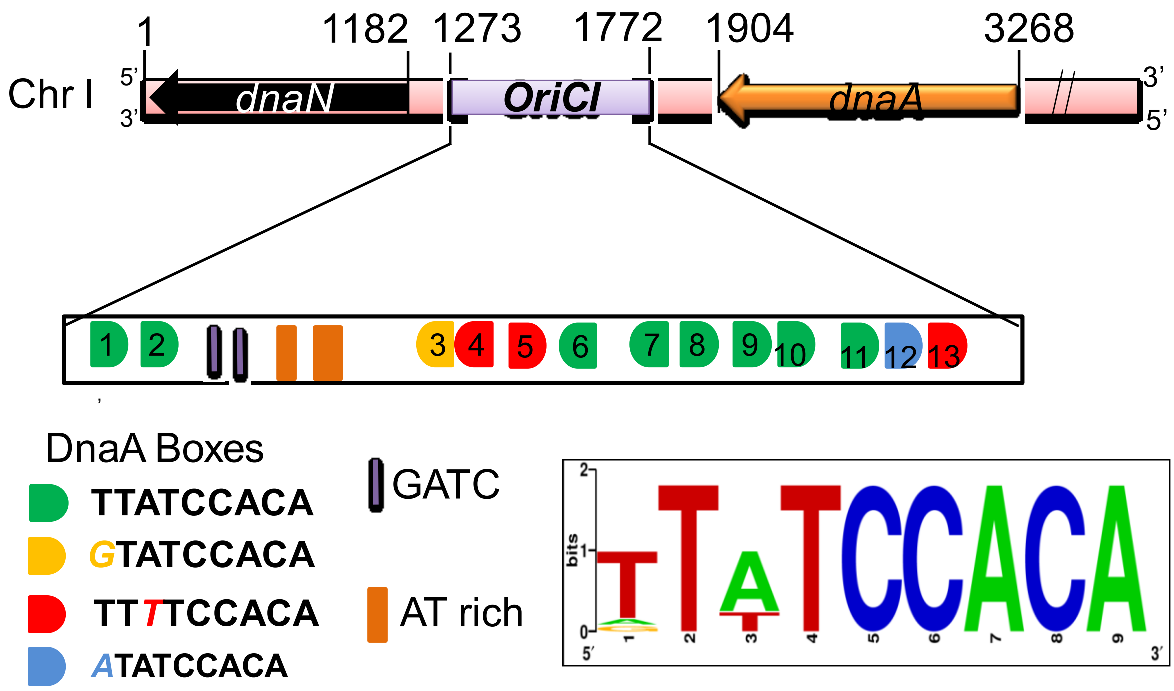

Fig S2. Organization of putative origin of replication in chromosome I (*oriCI*) of *D. radiodurans.* A *cis*-element region (1273-1772) located between *dnaN* and *dnaA* in chromosome I of *D. radiodurans* was analyzed for putative *oriC* signature. This fragment is found containing 13 copies of conserved DnaA boxes, 2 GATC motif and 2 AT-rich regions and thus predicted to be *oriCI in D. radiodurans*.

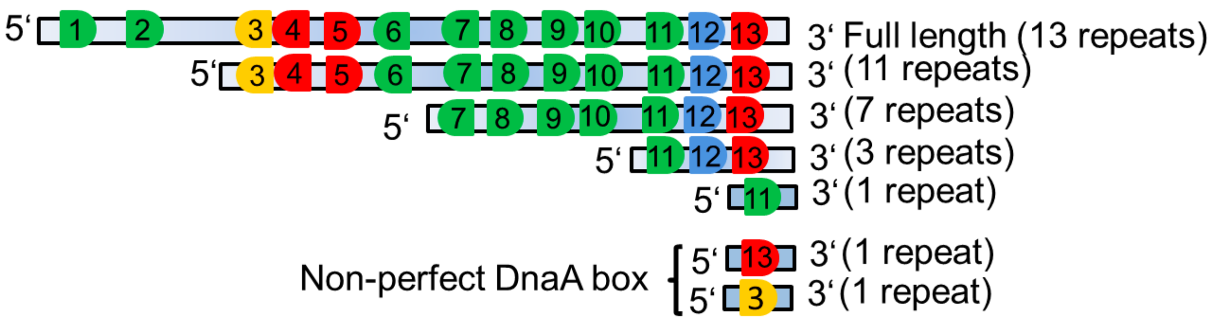

Fig. S3. Schematic representation of origin of replication structure in chromosome I (*oriCI*) and their repeat variants used for DNA protein interaction studies.

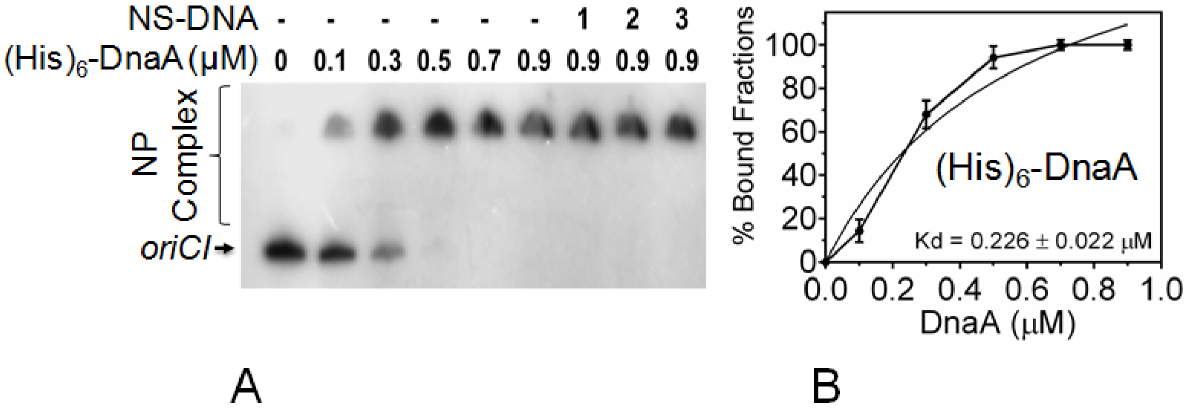

Fig. S4. Evaluation of histidine tag effect on DNA binding activity of DnaA. The different concentration of recombinant DnaA purified with histidine tag (His)6-DnaA) (A, B) was incubated with non-specific DNA (NS-DNA). Nucleoprotein complexes were resolved in PAGE and autoradiograms (A) were developed. Fraction of DNA in bound and free form was estimated densitometrically and plotted as a fraction of protein concentration (B).

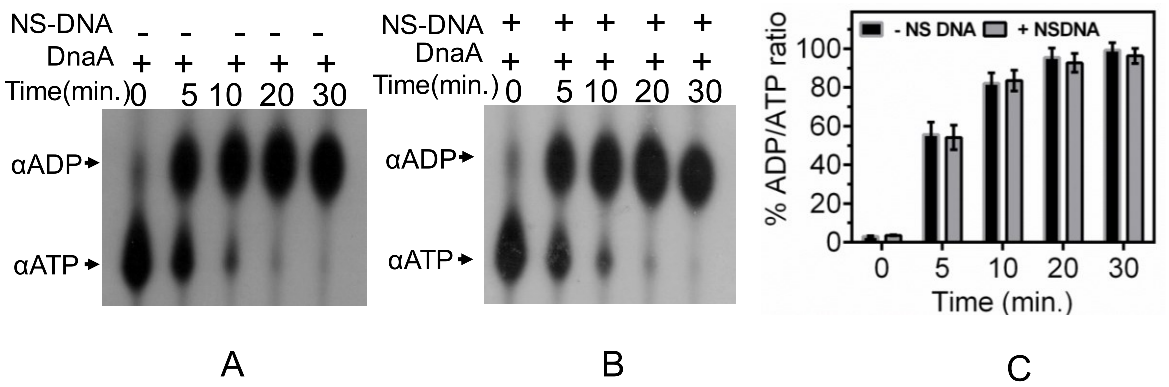

Fig. S5. Effect of non-specific DNA on ATPase activity of drDnaA. 2 μM concentration of drDnaA (DnaA) was incubated with [^{32}P]-αATP (αATP) at different time interval in the absence (A) and presence (B) of non-specific DNA (NS-DNA) and the generation of [^{32}P]-αADP (αADP) product was estimated. Percentage of ADP/ATP ratios were calculated and plotted as mean ± SD (n=3) (C). Results were analyzed using student t-test and significant difference in data set with p values of 0.05 or less is marked with (*) if any.

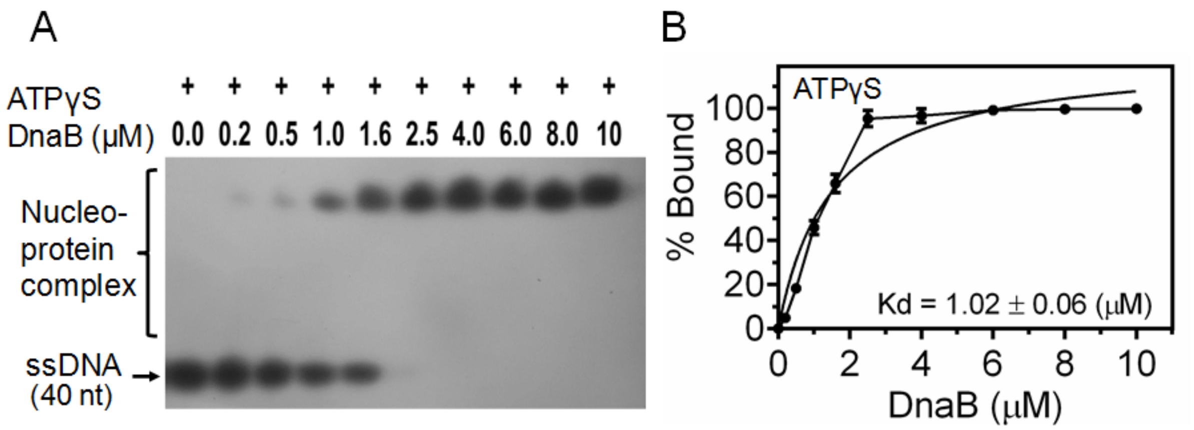

Fig S6. Effect of ATP hydrolysis on drDnaB binding to ssDNA. The affinity of deinoccoal DnaB was checked with 40 bases long ssDNA in the presence of non-hydrolysable analog of ATP (ATPγS). The levels of ssDNA bound to different concentration of drDnaB was estimated densitometrically and fraction of DNA bound to protein was plotted as a function of protein concentration. The Kd was determined using Graphpad Prism 6 software.

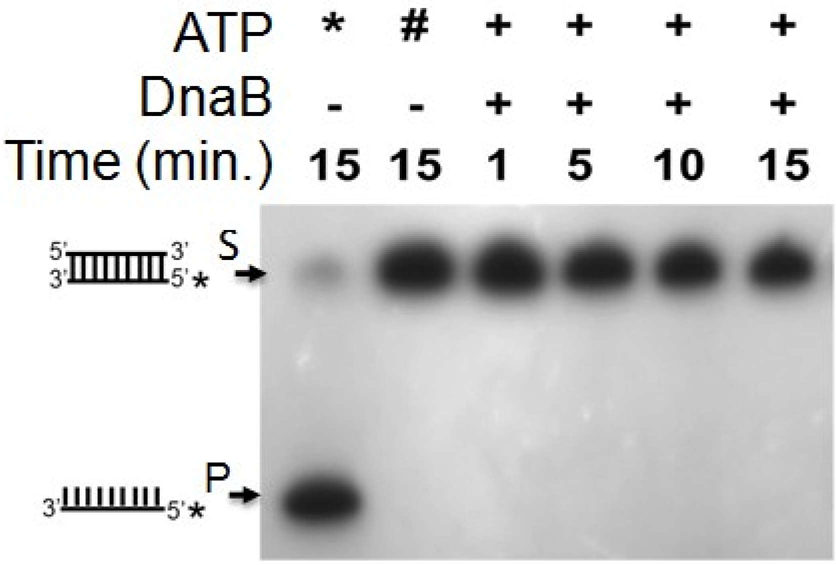

Fig S7. DnaB helicase activity with blunt end dsDNA substrate. The 2 μM recombinant DnaB protein was incubated with radiolabeled 37 bp blunt DNA for different time intervals . Mixtures were analysed on native PAGE and unwinding of dsDNA into single strand was compared with boiled dsDNA (*) and un-boiled DNA (#) as loading controls.

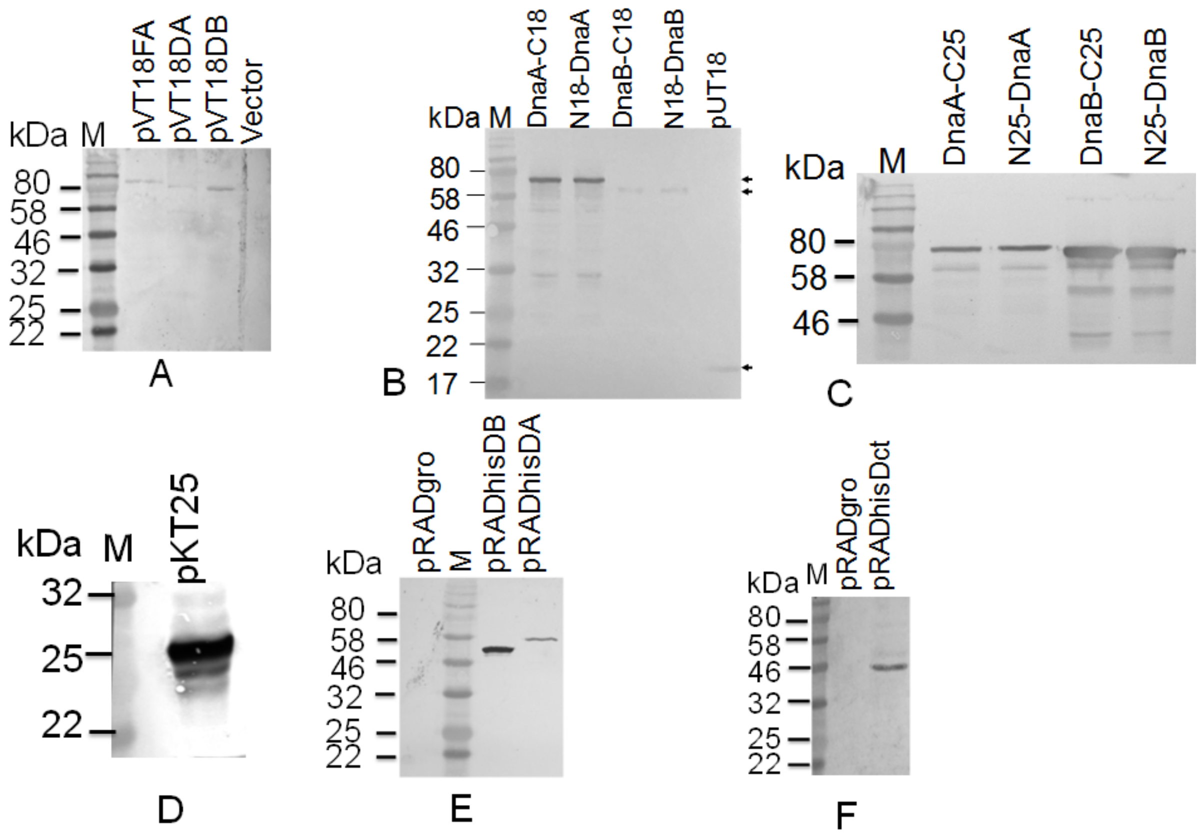

Fig. S8. Confirmation of the expression of T18 and T25 fusions of different proteins in *D. radiodurans* (A) surrogate *E. coli* BTH101 (B-D). Expression of polyhistidine tagged fusion of different proteins in *D. radiodurans* (E, F). Fusions were immunoblotted using antibodies against T18 (A,B), T25 (C,D), polyhistidine (E,F).

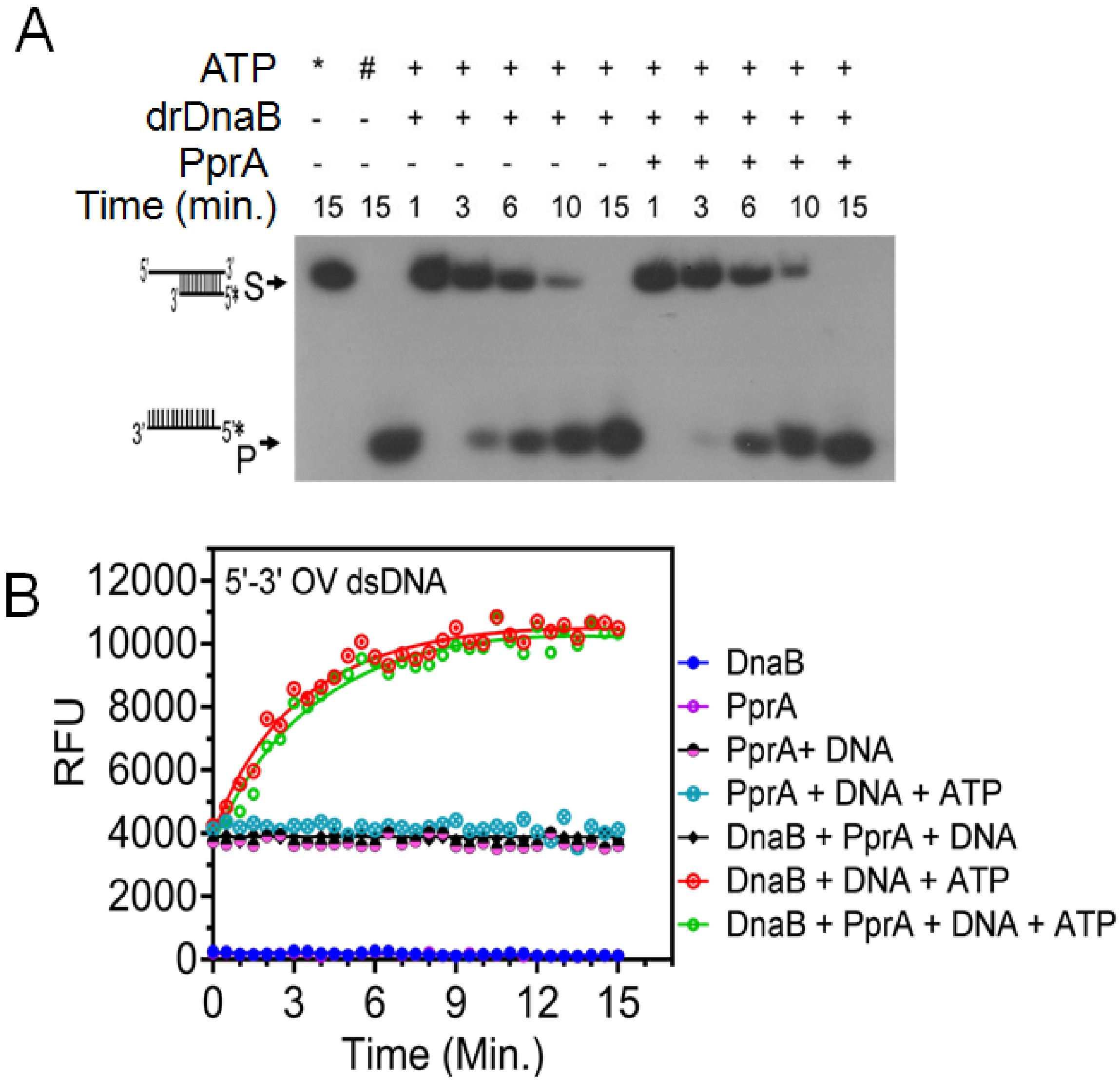

Fig S9. Effect of purified PprA on helicase activity of drDnaB. Recombinant drDnaB (DnaB) was incubated with radiolabeled dsDNA having 5' overhangs for the different time intervals in the presence of different combinations of PprA and ATP. The products were analyzed on denaturing PAGE (A). Similarly, FRET substrate of dsDNA with 5' overhang (Fig 9D) was incubated with drDnaB (DnaB) in different combinations as described in A. Helicase activity was monitored as the loss of FRET and gain of FAM emission with time (B). Data given are representative of the reproducible experiments repeated 2 times.

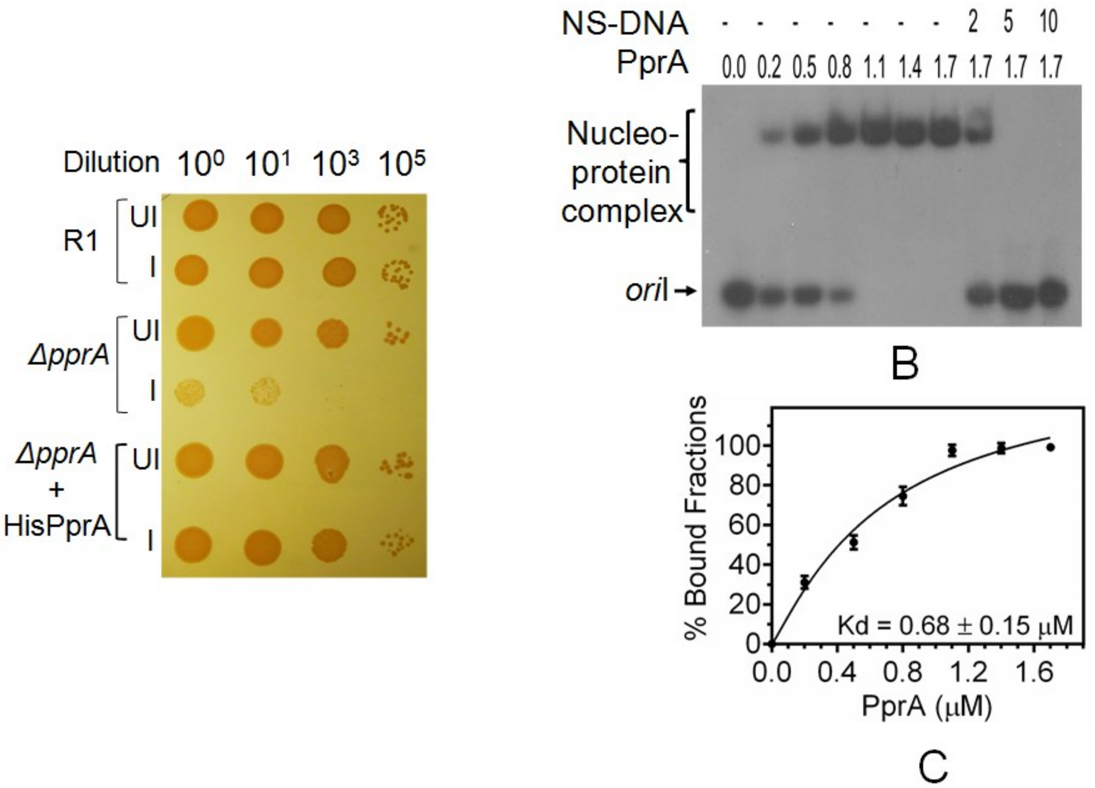

Fig. S10. Histidine tagged PprA complementation in *pprA* mutant and its binding to *oriCI*. Wild type *D. radiodurans* R1 (R1), *ΔpprA* mutant and mutant cells complemented with histidine tagged PprA expressing on pVHhisPprA [*ΔpprA*+HisPprA]) were irradiated with 6kGy gamma radiation (Dose rate 1.33 kGy/hr). Both irradiated cells (I) and corresponding SHAM controls (UI) were diluted and spotted on TYG agar plate (**A**). Different concentrations (0- 1.7 μM) of recombinant His-PprA (PprA) was incubated with radiolabeled *oriCI*. Saturated reaction mixture at 1.7 μM was chased with 2-fold (2), 5-fold (5) and 10-fold (10) higher molar concentrations of non-specific DNA (NS-DNA). Products were analyzed on non-denaturing PAGE and autoradiograms were developed **(B).** Band intensity of free and bound form of DNA substrate was measured densitometrically and mean ± SD (n=3) of percent bound fraction was plotted (C).

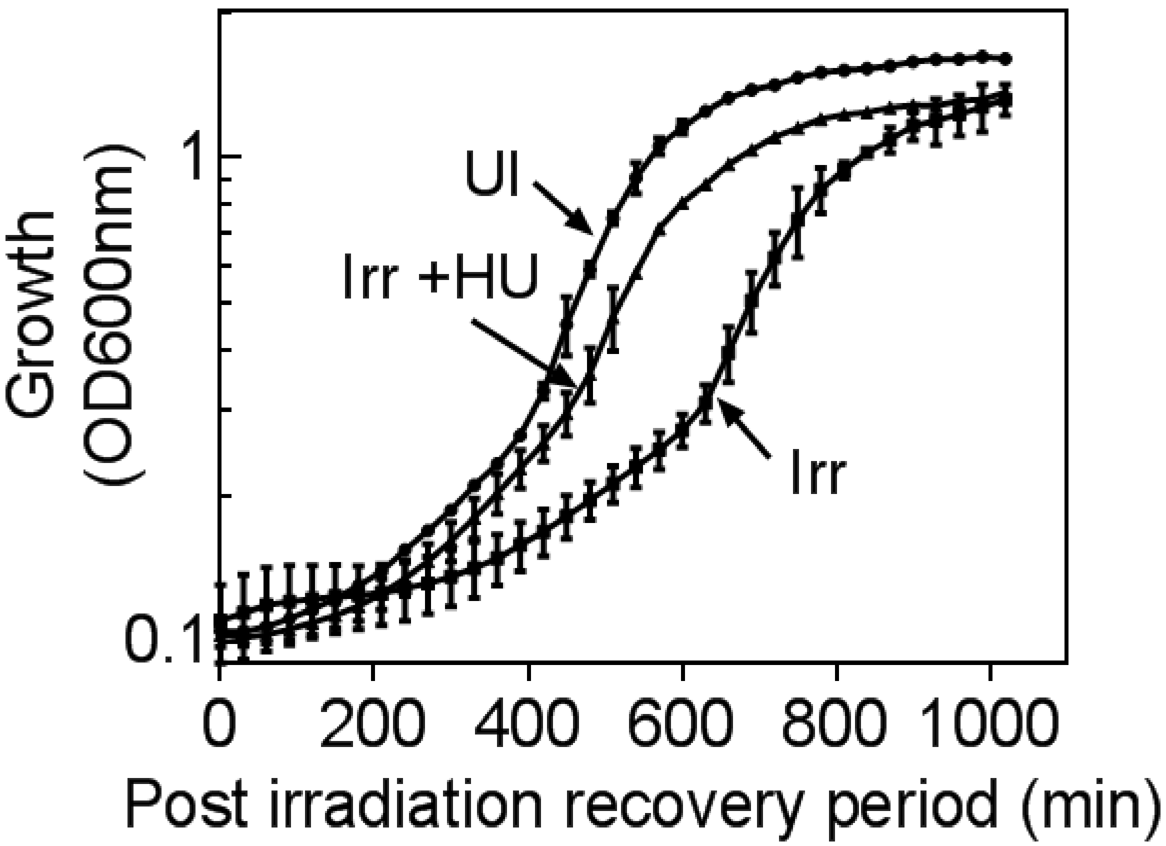

Fig S11. Effect of hydroxyurea on gamma radiation response of *D. radiodurans.* The *D. radiodurans* cells were treated with gamma radiation (Irr) in the presence of hydroxyurea (Irr+HU) and growth was compared with unirradiated and irradiated controls.

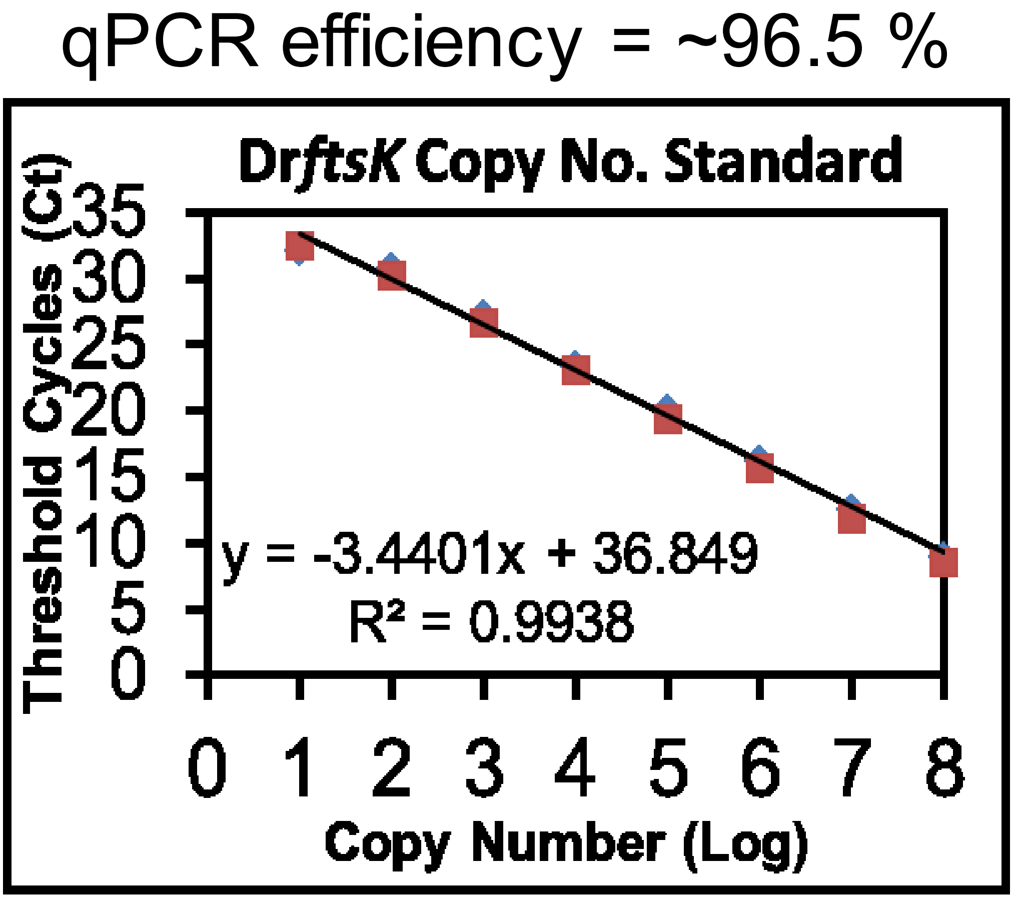

Fig. S12. Standard curve for the determination of copy number by RT-PCR. ~300 bp fragment of dr*ftsK* gene was PCR amplified and the known concentrations were taken for qPCR. The obtained Ct value for respective copy number was plotted to give standard curve.

Table S1 – List of bacterial strains and plasmids used in this study

Bacterial strains	Genotype	Source
Deinococcus radiodurans R1	Wild type strain ATCC13939	Lab Stock
ΔpprA mutant	*pprA* gene replaced with chloramphenicol resistance gene cassettes (Cam^R)	Lab Stock
E. coli NovaBlue	*end*A1 *hsd*R17*(r* $_{K12}{}^{-}$ *m* $_{K12}{}^{+}$*)* *sup*E44 *thi-1* *rec*A1 *gyr*A96 *rel*A1 *lac*F''*[pro*A^+B^+ *lac*I^q *Z*ΔM15*::*T*n*10] (Tet^R)	NEB Inc.,
E. coli BTH 101	F^-, *cya*-99, *ara*D139, *gal*E15, *gal*K16, *rps*L1 (Str^r), *hsd*R2, *mcr*A1, *mcr*B1	Karimova *et al.,* 1998
E. coli BL21(DE3) pLysS	F–, ompT, hsdS$_B$ (r$_B$–, m$_B$–), dcm, gal, λ(DE3), pLysS, Cm^r.	Lab stock

Plasmids

Sr No.	Plasmids	Characteristics	Sources	MW of Protein (~kDa)
1	pET28a(+)	~ 5.3 kb plasmid; N-terminal 6XHis tag (Kan^R)	Novagen	-
2	pETDnaA	pET28a(+) carrying Dr_0002 at *Bam*HI and *Eco*RI	This study	~ 53 kDa
3	pETDnaB	pET28a(+) carrying Dr_0549 at *Bam*HI and *Eco*RI	This study	~ 50 kDa
4.	pETDAΔCt	pET28a(+) carrying Dr_0002 without domain IV at *Bam*HI and *Eco*RI	This study	~ 40 kDa
5.	pETpprA	pET28a(+) carrying *pprA* at *Bam*HI and *Hin*dIII	Kota and Misra, 2006	~32 kDa
6	pUT18	pUC19 derivative, MCS at N-terminal of T18 fragments of adenylate cyclase, ~3 kb, AmpR	Karimova et al., 1998	~18 kDa
7	pUT18C	pUC19 derivative, MCS at C-terminal of T18 fragments of adenylate cyclase, ~3 kb, AmpR	Karimova et al., 1998	~18 kDa
8	pKNT25	pSU40 derivative, MCS at N-terminal of T25 fragment of adenylate cyclase, ~3.4 kb, KanR	Karimova et al., 1998	~25 kDa
9	pKT25	pSU40 derivative, MCS at C-terminal of T25 fragment of adenylate cyclase, ~3.4 kb, KanR	Karimova et al., 1998	~25 kDa
10	pUTEFA	pUT18 carrying *E. coli ftsA* at *Bam*HI and *Kpn*I	Modi and Mishra, 2014	~63 kDa
11	pKNEFZ	pKNT25 carrying *E. coli ftsZ* at *Bam*HI and *Kpn*I	Modi and Mishra, 2014	~65 kDa
12	pRADgro	pRAD1 carrying 261bp *Bgl*II-*Xba*I fragment of promoter (Pgro) from *D. radiodurans*	Misra et al., 2006	-
13	pRADhisDnaA	pRADgro carrying N-terminal 6XHis tagged *dnaA* from pETDnaA at *Apa*I-*Xba*I	Maurya et al., 2019b	~ 53 kDa
14	pRADhisDACt	pRADgro carrying N-terminal 6XHis tagged dr*dnaA* without domain IV from pETDAΔCt at *Apa*I-*Xba*I	This study	~40 kDa
15	pRADhisDB	pRADgro carrying N-terminal 6XHis tagged	This study	~50 kDa

		dr*dnaB* from pETDnaB at *Apa*I-*Xba*I		
16	pKNTDA	pKNT25 carrying *drdnaA* at *Bam*HI and *Eco*RI	Maurya et al., 2019b	~78 kDa
17	pKTDA	pKT25 carrying *drdnaA* at *Bam*HI and *Eco*RI	This study	~78 kDa
18	pUTDA	pUT18 carrying *drdnaA* at *Bam*HI and *Eco*RI	This study	~71 kDa
19	pUTCDA	pUT18C carrying *drdnaA* at *Bam*HI and *Eco*RI	This study	~71 kDa
20	pKNTDB	pKNT25 carrying *drdnaB* at *Kpn*I and *Eco*RI	Maurya et al., 2019b	~75 kDa
21	pKTDB	pKT25 carrying *drdnaB* at *Kpn*I and *Eco*RI	This study	~75 kDa
22	pUTDB	pUT18 carrying *drdnaB* at *Kpn*I and *Eco*RI	This study	~68 kDa
23	pUTCDB	pUT18C carrying *drdnaB* at *Kpn*I and *Eco*RI	This study	~68 kDa
24	pUTpprA	pUT18 carrying *pprA* at *Bam*HI-*Kpn*I	Kota et al., 2014	~50 kDa
25	pVHS559	An *E. coli – D. radiodurans* shuttle plasmid (SpecR)	Lab Stock	-
26	pSpecpprA	p11559 carrying *pprA* gene at *Nde*I-*Xho*I	Rajpurohit and Misra, 2013	~32 kDa
27	pV18DA	p11559 carrying T18 tagged dr*dnaA* gene from pUTDA at *Nde*I-*Xho*I	This study	~81 kDa
28	pV18DB	p11559 carrying T18 tagged dr*dnaB* gene from pUTDB at *Nde*I-*Xho*I	This study	~78 kDa

Table S2- List of Primers used in this study.

Primer	Oligonucleotide Sequences	Purpose
pETdnaBF	5’GGGGATCCATGGAAACGACTCCGCGT3’	pETDnaB
pETdnaBR	5’CGGAATTCTCACATCCCCTCCGGCGCCA3’	
pETDAF	5’CGGGATCCGTGCGCAAAAACGTCTC 3’	pETDAΔCt
pETDAΔCtR	5’CGGAATTCTCACATCTCGACCTTGGC3’	
BTDnaAF	5’CGGGATCCGGCAGCTGTGCGCAAAAACGTCTC3’	pUT18DA, pUT18CDA & pKTDA
BTDnaAR	5’GCGAATTCGCGGCCGCCGCCCCGACTTCTTC 3’	
BTDnaAR+	5’GCGAATTCTTACGCCCCGACTTCTTC 3’	
BTDnaBF	5’GGGGTACCGGCAGCTATGGAAACGACTCCGCGT3’	pUT18DB, pUT18CDB & pKTDB
BTDnaBR	5’CGGAATTCGCGGCCGCCATCCCCTCCGGCGCCA3’	
BTDnaBR+	5’CGGAATTCTTACATCCCCTCCGGCGCCA3’	
BTHF(PV)	5’GGAATTCCATATGACCATGATTACG 3’	pV18DA & pV18DB
BTHR(PV)	5’GGCCTCGAGCATATTACTTAGTTA3’	
pETHisF	5’AAAAGTACTGGGCCCATGGGCAGCAGCCAT3’	pRADhisDA, pRADhisDACt , pRADhisDB
pETHisR	5’CGCTTAAGTCTAGATATCTCAGTGGTGGTG3’	
RTZ Fw	5'ATCAAGGAATATCTCGA3'	qPCR primers
RTZ Rw	5'CAGCTTTTCGTTGTTCACC3'	
RTEFw	5’TTGAGCGTCCCCGCGCC3’	
RTERw	5’GTGCCCGACGAGGTAGA3’	
A155RTFw	5’TTGGCGCATTTTCCCGGC3’	
A155RTRw	5’CAGCAGGTTGATCGCCTG3’	
A0002RTFw	5’ACCCGGCGTCGTCCAGAG3’	
A0002RTRw	5’ GACGACCGGAACTTCAGC 3’	
B03RTFw	5’CTGAGTCCTGACGAGTCC3’	
B03RTRw	5’TTCCGGGTGACGCAGCAG3’	
B076RTFw	5’ATGAGTCCCCCCCTTGCC3’	
B076RTRw	5`CGGCGCGATCAACTCCAG3’	
C01RTFw	5’ATGTGCTCGCCTCCTAGA3’	
C01RTRw	5’ TCACTGTGAAACCTGATC3’	
C18RTFw	5’ATGACACAGACGCGGCG3’	
C18RTRw	5’GTCCGCGAGGCGCATCAT3’	
OriIFw	5’CGGGATCCGTTTTTGCAGCCAACTCCCGA3’	Primers used for generation of different number of DnaA boxes for EMSA with drDnaA
OriIRw	5’GCGAGCTCCCGTTCCAAATGGCGGTGA3’	
OriIF1	5’CGGGATCCGTAGTAGCAGTAG3’	
OriIF2	5’CGGGATCCCGAAAACCTGATG3’	
OriI3Fw	5’AACTTATCCACAGGATATCCACAGGTTTTTCCACAGA3’	
OriI3Rw	5‘TTCTGTGGAAAAACCTGTGGATATCCTGTGGATAAGT3’	
OriI4.1Fw	5’GGGTTATCCACAGGGC3’	
OriI4.1Rw	5’GCCCTGTGGATAACCC3’	
OriI4.2Fw	5’GGGTTTTCCACAGGGC3’	
OriI4.2Rw	5’GCCCTGTGGAAAACCC3’	
OriI4.3Fw	5’GGGGTATCCACAGGGC3’	

OriI4.3Rw	5’GCCCTGTGGATACCCC3’	
Linear 3OVFAM	[FAM]5’GCACTGGCCGTCGTTTTACTCGTGACTGGGAGACCC3’	FRET substrate 3’ or 5’ overhang Linear dsDNA
Linear 5OVFAM	[FAM]5’GCA CTG GCC GTC GTT TTA CTC GTG3’	
BHQ-comp_ linear OV	5’TCCAGTCACGAGTAAAACGACGGCCAGTGC3’[BHQ1a~Q]	
TRAP comp linear OV	5’TACGAGTAAAACGACGGCCAGTGC3’	FRET TRAP
500bpFw	5’GGAATTCCATATGCAAGCAGCCAGAATTCGCGT 3’	PCR amplification of non-specific DNA
500bpRw	5'CAGCTTTTCGTTGTTCACC3' (RTZRw)	
260bpFw	5'ATCAAGGAATATCTCGA3' (RTZFw)	
260bpRw	5'CAGCTTTTCGTTGTTCACC3' (RTZRw)	
170bpFw	5'ATCAAGGAATATCTCGA3' (RTZFw)	
170bpRw	5’CGCATGCGCTTGGGGCCT3’	
37bpFw	5' CGGAATTCGCGGCCGCGATTTTTTCGACACGTTGCAG 3’ (DRparA1R(-SC)	
37bpRw	5’CTGCAACGTGTCGAAAAAATCGCGGCCGCGAATTCCG3’	
16bpFw	5’CCGAGGAAAACGTCCA3’	
16bpRw	5’ TGGACGTTTTCCTCGG3’	
